# Genome wide association studies reveal candidate genes for salt tolerance in safflower (*Carthamus tinctorius* L.) at seedling stage

**DOI:** 10.3389/fpls.2026.1630492

**Published:** 2026-03-06

**Authors:** Fawad Ali, Obaid Ullah Shah, Muhammad Azhar Nadeem, Muhammad Tanveer Altaf, Arif Ali, Mian Abdur Rehman Arif, Emre Aksoy, Faheem Shehzad Baloch

**Affiliations:** 1 School of Breeding and Multiplication (Sanya Institute of Breeding and Multiplication), School of Tropical Agriculture and Forestry, Hainan University, Sanya, China; 2 Department of Plant Resources and Environment, Jeju National University, Jeju, Republic of Korea; 3 School of Agriculture and Biology, Shanghai Jiao Tong University, Shanghai, China; 4 Department of Biotechnology, Faculty of Science, Mersin University, Mersin, Türkiye; 5 Department of Biotechnology, Samarkand State University, Samarkand, Uzbekistan; 6 Department of Field Crops, Faculty of Agriculture, Recep Tayyip Erdoğan University, Rize, Türkiye; 7 Department of Plant Sciences, Quaid-i-Azam University, Islamabad, Pakistan; 8 Nuclear Institute for Agriculture and Biology College, Pakistan Institute of Engineering and Applied Sciences (NIAB-C, PIEAS), Faisalabad, Pakistan; 9 Department of Biological Sciences, Middle East Technical University, Ankara, Türkiye; 10 Sh. Rashidov Samarkand State University, Institute of Biochemistry, Department of Genetics, Samarkand, Uzbekistan

**Keywords:** candidate genes, in silico analysis, MTAS, oilseed crop, PPI network, salt stress

## Abstract

Safflower productivity is hindered by soil salinity, making the identification of genetic markers essential for breeding resilient cultivars. Despite the substantial yield losses caused by salt stress, research on parental genotypes and candidate genes associated with salt tolerance remains limited. A pot experiment with 94 safflower genotypes exposed to four sodium chloride (NaCl) concentrations at the seedling stage explored salt tolerance genetics. Results showed significant variability among genotypes, NaCl treatments, and their interactions for most traits, except biological yield (BY) and fresh shoot weight (FSW). Traits showed reductions from 8% (number of leaves) to 76% (dry root weight) under NaCl stress. Broad sense heritability ranged from 17% to 97%. Correlation analysis revealed positive associations among traits, except FSW and BY. PCA grouped genotypes into three distinct clusters. Using stress tolerance indices (> 0.65) and superior performance above the population mean, three top-performing safflower genotypes were identified. A genome-wide association study (GWAS) revealed 322 marker-trait associations (MTAs), distributed as follows: 34 for BY, 25 for dry root weight (DRW), 44 for dry shoot weight (DSW), 48 for fresh root weight (FRW), 46 for FSW, 60 for number of leaves (NL), 47 for plant height (PH), and 18 for root length (RL). Gene annotation revealed key candidates influencing salinity tolerance, including *PLA1*, *APK4*, *GINT1*, *TPLATE*, *UL13M*, *SPP2*, *FRF3*, *AT1G33770*, *AT5G01610*, *DTX50*, and *RAF1*. These genes regulate sulfation of secondary metabolites, chloroplast development, site-specific cell wall modifications, sucrose biosynthesis, and calcium signaling, as well as the functions of hypothetical proteins or proteins with unknown roles. Validating these candidate genes, *in silico* transcriptomics showed significant upregulation of *PLA1*, *SPS2*, and *DTX50*, alongside downregulation of *APK4*, *GINT1*, *TPLATE*, *UL13M*, *FRF3*, *AT1G33770*, *AT5G01610*, and *RAF1* under salinity. These findings highlight the top-performing genotypes for salt-tolerant cultivar development and warrant further functional studies on the identified candidate genes to gain a deeper understanding of their mechanisms under salt stress.

## 1 Introduction

Safflower (*Carthamus tinctorius* L.) is an autogamous oilseed crop in the Compositae family, characterized by a haploid genome of size 1.4 GB and 24 chromosomes (2n = 24) (Kumari et al., 2017). This versatile crop is grown globally for diverse applications, including dye production, culinary oil extraction, and medicinal purposes (Weiss, 2000). Safflower, a moderately salt-tolerant crop, faces productivity challenges due to various threats including human activities, climate change, and unfavorable environmental conditions (Yeilaghi et al., 2012). Soil salinity, a consequence of climate change, is the second most significant abiotic factor affecting global agricultural productivity, impacting numerous physiological, biochemical, and molecular processes (Raza et al., 2023). Although safflower can grow in salt-affected areas where many other plants cannot survive, its yield may still be reduced when exposed to salt stress (Yeilaghi et al., 2012). Salt-affected safflower plants typically exhibit smaller size, increased succulence, thickened and darker green leaves, reduced stem diameter and plant height, lowered transpiration rates, altered leaf cell structure, and fewer stomata (Hussain et al., 2016). Salinity can hasten flowering and maturity, and reduce the number of capitula per plant and seeds per capitula, particularly in tertiary heads. Excessive accumulation of neutral soluble salts in the rhizosphere can disrupt nutrient uptake, further impacting plant growth and development (Hussain et al., 2016). Given these challenges, there is a pressing need to identify salinity-responsive genotypes and genes to develop salt-tolerant safflower varieties. Research has shown that vigorous seedling growth and rapid seed germination play a crucial role in plant establishment and yield potential. Consequently, the germination and seedling stages are widely recognized as ideal time points for screening germplasm collections to assess salt stress tolerance (Xu et al., 2024). For example, Thoday-Kennedy et al. (2021); Shaki et al. (2019), and Fatahiyan et al. (2025) emphasized screening safflower germplasm at the seedling stage, while Xu et al. (2024) evaluated wheat germplasm for salt stress tolerance at the seedling stage.

Salt tolerance in plants is a multifaceted trait involving numerous genes, intricate regulatory networks, complex signal transduction pathways, and diverse metabolic processes (Devi et al., 2019). The response of crops to saline conditions is highly variable, depending on factors such as stress duration, intensity, genotype, and growth stage (Akram et al., 2022). This complexity makes it challenging to evaluate salt tolerance in genetic panels, particularly during the seedling stage. To advance the development of salt-tolerant crop varieties, it is essential to gain a deeper understanding of the molecular mechanisms underlying salinity tolerance-related traits. GWAS have emerged as a powerful genetic tool to unravel these complex relationships and identify key genetic factors contributing to salt tolerance (Zhang et al., 2023) and other abiotic stresses in crop plants (Arif et al., 2012, Arif et al., 2017).

Previous studies have provided experimental evidence for QTLs and candidate genes associated with seedling salt tolerance across various plant species. In maize, a GWAS using 557,894 polymorphic SNPs on 348 inbred lines identified 13 candidate genes linked to seedling salt tolerance, with *ZmPMP3* and *ZmCLCg* confirmed as key genes. Notably, *ZmCLCg* was found to function as a chloride transporter in maize (Luo et al., 2021). Cotton research utilizing 18,430 polymorphic SNPs across 149 genotypes revealed six genes involved in seedling salt tolerance: *Gh_D08G1309, Gh_D08G1308, Gh_A01G0908, Gh_A01G0906, Gh_D01G0945*, and Gh_*D01G0943*. These genes were associated with various cellular processes, including cell amplification, auxin response, N-glycosylation, transmembrane transport, osmotic pressure balance, sucrose synthesis, and intracellular transport (Zheng et al., 2022). In barley, a study of 121 accessions using 9K SNPs uncovered approximately 1500 candidate genes, with potassium channel-encoding genes mapped to chromosome 1H. Additionally, the squamosa promoter-binding-like protein 6 on chromosome 5H was linked to seedling salt tolerance (Thabet et al., 2021). Rice research involving 203 accessions led to the identification of 26 QTLs associated with seedling salt tolerance. Promising candidate genes within these QTLs included those encoding glycosyl hydrolase, sucrose transporter, leucine zipper transcription factor, ammonium transporter, and MYB transcription factor (Batayeva et al., 2018). These findings collectively demonstrate the complex genetic basis of seedling salt tolerance across different crop species and highlight potential targets for improving salt stress resilience in plants.

Genetic diversity for salt tolerance traits is essential for developing salt-tolerant cultivars. While QTLs associated with salt tolerance have been extensively mapped in crops such as wheat using diverse germplasm panels, there remains limited information on MTAs for salt response in safflower identified through GWAS. Therefore, identifying genotypes and underlying QTLs is critical in safflower breeding programs focused on enhancing salt tolerance to ensure high performance under saline conditions. To address this gap, this study targeted to evaluate a collection of genotypes and uncover putative candidate genes for salt tolerance at the seedling stage in safflower employing the *Silico-DArT* marker information. The primary objective was to screen the safflower germplasm panel for salt stress tolerance based on morphological traits and to pinpoint candidate genes for future marker-assisted breeding programs. The tolerant genotypes and identified candidate genes may prove valuable for developing salt-tolerant cultivars.

## 2 Materials and methods

### 2.1 Soil analysis

The experimental setup for cultivating safflower genotypes utilized a soil mixture consisting of sand and soil in a 1:2 ratio, which was used to fill the pots. A comprehensive soil analysis was conducted, with micro nutrients assessed using atomic absorption and macro nutrients evaluated via flame photometry. Each pot contained 780 g of soil that had been passed through a 4 mm sieve. The pots were placed in a glasshouse environment, maintaining a temperature range of 26-28 °C and 46% humidity. The experiment was conducted under a light cycle of 16 hours followed by 8 hours of darkness. Initially, three seeds of each safflower genotype were planted in each pot, with subsequent thinning after germination to retain only one seedling per pot for data collection. The plants were watered regularly with tap water, adjusting the frequency based on the specific requirements of each genotype.

### 2.2 Plant materials and evaluation

The study utilized 94 safflower genotypes sourced from 26 countries, procured from the United States Department of Agriculture (USDA) (**Supplementary Table 1**). The experiment, initiated on February 10th, 2024, employed a two-factorial randomized complete block design with three replications and four treatments. The experiment involved three plants per genotype for data collection.

The four NaCl treatments included: T1 (control with no NaCl), T2 (80 mM NaCl), T3 (160 mM NaCl), and T4 (250 mM NaCl). These treatments were selected based on established literature for safflower salt stress studies and preliminary experiments conducted in our lab (Thoday-Kennedy et al., 2021). These concentrations span control to severe stress levels commonly applied in safflower phenotyping, allowing discrimination of tolerant genotypes while avoiding complete lethality. 250 mM concentration simulates extreme field-relevant salinity, within safflower’s moderate tolerance range but sufficient to identify resilient genotypes without universal mortality, consistent with hydroponic and soil-based assays (Thoday-Kennedy et al., 2021). The NaCl applications in T2, T3, and T4 were administered between 16 and 35 days after sowing, spread over several days to prevent salt shock. At 35 days post-sowing, the safflower genotypes were harvested, and key morphological traits were recorded. For the association analysis, the mean values of each trait across all treatments were utilized.

### 2.3 Studied parameters

Plant height (PH) was determined by measuring from the stem base to the shoot apex in centimeters with the help of a scale. The number of leaves (NL) were counted on each selected plant manually. The same plant was then weighed to record its biological yield (BY) in grams. For root length (RL) measurement, each plant was carefully uprooted and washed with tap water. RL was measured in centimeters with the help of a scale. Fresh root (FRW) and shoot (FSW) weights were recorded in grams using a digital balance. The fresh shoots and roots of the selected plants were oven-dried and then weighed to measure the dry shoot (DSW) and root (DRW) weights.

### 2.4 DNA extraction

DNA was extracted from two-week-old safflower seedlings using two methods: the CTAB protocol as described by Doyle and Doyle in 1990 (Doyle and Doyle, 1990), and a specific protocol recommended by Diversity Arrays Technology. The latter protocol is available on the Diversity Arrays Technology website (https://www.diversityarrays.com/services/laboratory-services/).

### 2.5 Statistical analysis

#### 2.5.1 Phenotypic screening and genotype evaluation

Descriptive statistics and ANOVA in Statistix 8.1 tested phenotypic variability and genotype and treatment effects. Broad sense heritability was estimated using the formula given below.


$${\text{H}}^{2}={\text{σ}}^{2}\text{G}/\left({\text{σ}}^{2}\text{G}+\left({\text{σ}}^{2}\text{G}\times \text{T}/\text{t}\right)+\left({\text{σ}}^{2}\text{e}/\left(\text{t}\times \text{r}\right)\right)\right)$$

$${\text{σ}}^{2}\text{G}=\left(\text{MS}\_\text{G}–\text{MS}\_\text{G}\times \text{T}\right)/\left(\text{t}\times \text{r}\right)$$

$${\text{σ}}^{2}\text{G}\times \text{T}=\left(\text{MS}\_\text{G}\times \text{T}–\text{MS}\_\text{e}\right)/\text{r}$$

$${\text{σ}}^{2}\text{e}=\text{MS}\_\text{e}$$

Where:

MS_G = Mean square of genotypes
MS_G×T = Mean square of genotype × treatment interaction
MS_e = Error mean square
t = number of treatments
r = number of replications

Data visualization used RStudio v1.0.153 with ggplot2 for boxplots (Wickham, 2016) and factoextra for correlation and PCA plots (Epskamp et al., 2012; Kassambara and Mundt, 2017). The stress tolerance index (STI; saline/control ratio) categorized genotypes as sensitive (<0.35), moderately sensitive (0.35–0.50), moderately tolerant (0.50–0.65), or tolerant (>0.65) (Tao et al., 2021). Superior genotypes (mean > population mean across treatments) were verified via iPASTIC (Ali et al., 2024).

#### 2.5.2 DArTseq markers analysis, population structure, and GWAS

All images generated by the DArTseq platform were analyzed using *DArTsoft v.7.4.7* (DArT P/L, Canberra, Australia). *SilicoDArT* markers, identified through DArTseq and scored in a binary format (presence = 1, absence = 0) as described by Baloch et al. (2017), were utilized for analysis. Marker quality was assessed based on call rate, polymorphism information content (PIC), and repeatability. Markers with PIC values below 0.10, repeatability under 100%, and call rates less than 0.80% were excluded to ensure reliable results and avoid false inferences.

Population structure analysis was previously conducted as described in Ali et al. (2020a). Genotyping was performed as described by Ali et al. (2024). The chromosome locations and physical positions of the markers across different chromosomes were obtained from the Saf_v1.gff3 file (Wu et al., 2021). MTA analysis was performed using TASSEL 5.0.5 with a mixed linear model (MLM, Q + K) approach (Bradbury et al., 2007), which incorporated Q-matrix (Q) and kinship (K) matrix to account for population and family structure, following the guidelines of Zia et al. (2020). The kinship matrix was calculated using scaled identity and descent methods implemented in TASSEL 5.0.5 (Bradbury et al., 2007). In the association study, the *p*-value indicated the strength of the marker-trait relationship, while R^2^ represented the percentage of phenotypic variance explained by significant markers (Jin et al., 2011). Significant *SilicoDArT* markers were identified based on Bonferroni and FDR thresholds of *p* = 0.01. For visualization, a Manhattan plot was generated using the “cMplot” R package in R 4.0.0 statistical software.

#### 2.5.3 Protein-protein interactions and gene ontology enrichment analysis

Marker sequences were BLAST searched against the *Arabidopsis thaliana* genome (TAIR 10) via TAIR BLAST 2.9.0+ (Swarbreck et al., 2007), and the loci with the highest hit score and E cut-off value of 0.001 were selected (Lamesch et al., 2012). The identified *Arabidopsis* orthologues were then used to construct protein-protein interaction (PPI) networks using String 12.0 (https://cn.string-db.org/), with a minimum interaction score of 0.9 (Szklarczyk et al., 2019). To enhance network reliability, a filtering process was applied using the ‘combined score’ with a recommended threshold of 400 (von Mering et al., 2005). The genes encoding proteins interacting with marker-associated loci in each PPI network were subsequently used for gene ontology (GO) enrichment analysis via PANTHER v.14 (Mi et al., 2019), employing Fisher’s exact test with Bonferroni correction for multiple testing (*P*< 0.01) (Thomas et al., 2022), and the top enriched GO terms were listed to provide a general understanding of potential functions of the PPI networks. Finally, GO enrichment for molecular function and biological process categories were visualized by String version 12.0 with a signal and a strength rate of ≥0.01.

#### 2.5.4 *In silico* transcriptomics analysis of *Arabidopsis* orthologs of the marker genes under salinity

Expression of the *Arabidopsis* orthologs of the marker genes were investigated in five transcriptomics studies. To this end, microarray datasets GSE71001 and GSE7642 were downloaded from Gene Expression Omnibus (GEO) (Clough and Barrett, 2016) and were analyzed by GEO2R tool (Edgar et al., 2002) with log_2_ fold change ≥ 2 and significance level P-value of 0.05 after quantile normalization to the expression data. At the end of these analyses, differentially expressed genes (DEGs) were determined in each microarray experiment. The FASTQ and SRA files of RNA sequencing datasets GSE229217 and GSE270516 were downloaded from GEO, and converted to read counts CVS files in Galaxy server using Salmon tool after quality control and pre-processing (Giardine et al., 2005) according to Batut et al. (2021). All RNA-seq datasets (GSE229217 and GSE270516) were processed using Salmon (Patro et al., 2017) with quasi-mapping against the Araport11 *Arabidopsis thaliana* reference transcriptome (Cheng et al., 2017). In addition to read count data used for DEG identification, TPM (Transcripts Per Million) values were also generated and used for expression comparison and heatmap clustering. To enable integration of expression data across these two platforms, all values were z-score normalized within each dataset before being merged into a single matrix for clustering. This approach reflects relative expression patterns, rather than raw TPMs or fold changes. Then, the read counts were pre-processed and analyzed by iDEP 2.0 (Ge et al., 2018) using EdgeR package (Robinson et al., 2010). Finally, DEGs were identified by DEseq2 with log_2_ fold change ≥ 2 and significance level P-value of 0.05. Adjustment to the P-values was done by Benjamini & Hochberg (False discovery rate - FDR) analysis (Benjamini and Hochberg, 1995). Six-week-old *Arabidopsis thaliana* Colombia-0 (Col-0) seedlings were treated with 150 mM NaCl for 4 hours and their roots were used in microarray analyses in GSE71001 (Hartmann et al., 2015), whereas five-day-old Col-0 seedlings were treated with 140 mM NaCl for 30 minutes, 1 hour, 4, 16 and 32 hours and their roots were used in microarray analyses in GSE7642 (Dinneny et al., 2008). Ten-day-old *Arabidopsis thaliana* Colombia-0 (Col-0) seedlings were grown in an MS solution including 150 mM NaCl for 8 hours and their roots and shoots were used in RNA-seq analyses in GSE229217 (Seok et al., 2023), while ten-day-old Col-0 seedlings were grown in ½ MS solution including 150 mM NaCl for 4 hours and their roots were used in RNA-seq analyses in GSE270516 (Bellucci et al., 2024). Finally, a heat map of the expression patterns was generated by “plotty” function in ggplot2 package in R following Euclidean distance hierarchical clustering (Wickham, 2011).

## 3 Results

### 3.1 Soil analysis

The soil composition comprised a blend of sand and soil, mixed in a proportion of one part sand to two parts soil. The concentrations of micro and macronutrients present in this mixture are detailed in **Table 1**.

**Table 1:** Soil analysis reported micro and macro nutrients content.

Micro nutrients							Macro nutrients (mg kg^-1^)				
pH	Eco mS m^-1^	Organic matter (%)	Available phosphorus (mg kg^-1^)	Available potassium (mg kg^-1^)	Saturation (%)	Texture	Zn	Fe	B	Mn	Cu
8.6	1.2	0.84	2.7	204	32	Loam	1.58	4.8	0.46	7.3	0.78

### 3.2 Phenotypic traits measurement and genotype evaluation

The analysis of variance (ANOVA) results indicated statistically significant variations among safflower genotypes, NaCl treatments (T), and their interaction (G * T) for the majority of traits examined. However, BY and FSW were exceptions, showing no significant differences in these analyses (**Table 2**).

**Table 2:** Analysis of variance showed highly-significant variation among genotypes (G), NaCl treatments (T), and their (G * T) interaction.

Trait	Source of variation	Mean squares	F value	P value
BY	G	10.117	1.04	0.373^ns^
	T	20.642	2.13	0.095^ns^
	G * T	9.579	0.99	0.537^ns^
DRW	G	0.008	11.31	0.0000****
	T	0.973	1385.99	0.0000****
	G * T	0.003	3.65	0.0000****
DSW	G	0.017	11.12	0.0000****
	T	0.984	635.51	0.0000****
	G * T	0.003	1.65	0.0000****
FRW	G	0.043	251.78	0.0000****
	T	1.754	10364.7	0.0000****
	G * T	0.002	10.44	0.0000****
FSW	G	1.876	1.02	0.434^ns^
	T	4.771	2.59	0.052^ns^
	G * T	1.864	1.01	0.442^ns^
NL	G	2.725	773.15	0.0000****
	T	51.389	14579.1	0.0000****
	G * T	0.574	162.78	0.0000****
PH	G	7.823	264.78	0.0000****
	T	172.866	5850.85	0.0000****
	G * T	0.571	19.34	0.0000****
RL	G	4.778	249.29	0.0000****
	T	114.719	5985.37	0.0000****
	G * T	0.374	19.52	0.0000****

**** showing the highly significance level at p < 0.0001 and ns shows non-significant.

**Figure 1:**
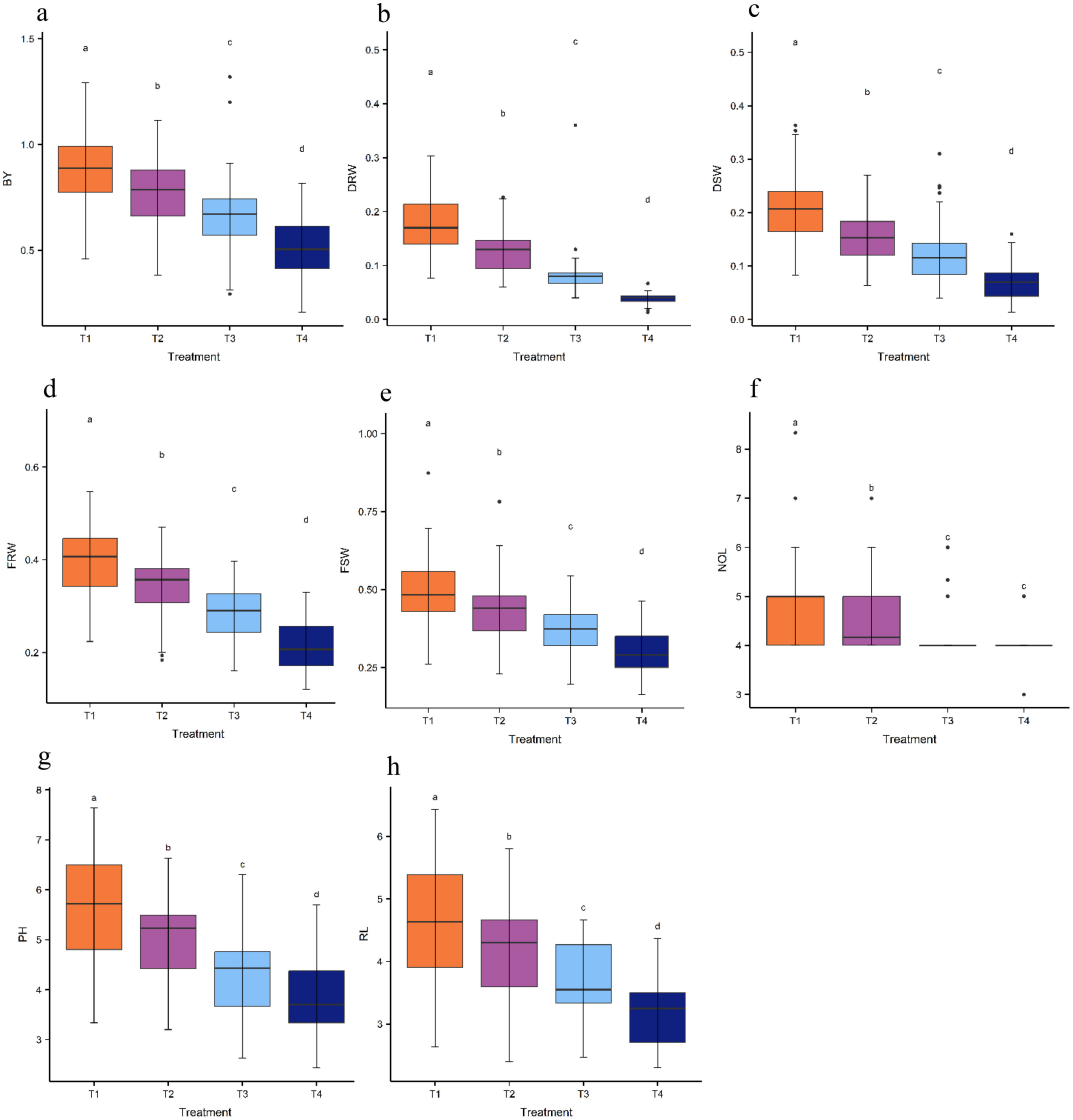
Boxplot analysis revealed significant differences among the treatments. **(a)** BY, biological yield, **(b)** DRW, dry root weight, **(c)** DSW, dry shoot weight, **(d)** FRW, fresh root weight, **(e)** FSW, fresh shoot weight, **(f)** NOL, number of leaves, **(g)** PH, plant height, **(h)** RL, root length.

The varying levels of NaCl had a significant impact on all the studied traits across the treatments (**Table 3**). For instance, PH decreased from 5.64 cm in T1 to 4.96, 4.36, and 3.82 cm in T2, T3, and T4, respectively. FRW declined from 0.40 g in T1 to 0.35, 0.28, and 0.22 g in T2, T3, and T4, respectively. FSW dropped from 0.49 g in T1 to 0.42, 0.37, and 0.30 g in T2, T3, and T4, respectively. NL decreased from 5.00 in T1 to 4.60, 4.20, and 4.05 in T2, T3, and T4, respectively. RL reduced from 4.62 cm in T1 to 4.15, 3.64, and 3.15 cm in T2, T3, and T4, respectively. Likewise, BY declined from 0.88 g in T1 to 0.77, 0.67 and 0.51 g in T2, T3, and T4, respectively. DSW decreased from 0.21 g in T1 to 0.16, 0.12, and 0.07 g in T2, T3, and T4, respectively, while DRW dropped from 0.17 g in T1 to 0.13, 0.08, and 0.04 g in T2, T3, and T4 (**Table 3**). Varying NaCl levels significantly affected the average performance of all the studied traits. Notable differences were observed among treatments for all traits, except for NL, where no differences were detected between T3 and T4 (**Figure 1**). The values of broad sense heritability ranged from 17% to 97% across the evaluated traits (**Table 4**).

**Table 3:** Range (minimum and maximum values) with mean ± standard deviation (S.D) values of the studied traits in safflower genotypes under varying NaCl treatments.

Treatment	T1		T2		T3		T4	
Trait	Range	Mean ± S.D	Range	Mean ± S.D	Range	Mean ± S.D	Range	Mean ± S.D
PH	3.33-7.63	5.64 ± 1.12	3.20-6.63	4.96 ± 0.86	2.63-6.30	4.36 ± 0.8	2.43-5.70	3.82 ± 0.74
FSW	0.26-0.87	0.49 ± 0.11	0.23-0.63	0.42 ± 1.57	0.20-0.54	0.37 ± 0.08	0.16-0.46	0.30 ± 0.07
NL	4.00-8.33	5.00 ± 0.86	4.00-7.00	4.60 ± 0.69	4.00-6.00	4.20 ± 0.43	3.00-5.00	4.05 ± 0.27
FRW	0.22-0.55	0.40 ± 0.08	0.18-0.47	0.35 ± 0.07	0.16-0.40	0.28 ± 0.06	0.12-0.33	0.22 ± 0.05
RL	2.63-6.43	4.62 ± 0.86	2.40-5.80	4.15 ± 0.77	2.47-4.67	3.64 ± 0.61	2.30-4.37	3.15 ± 0.51
BY	0.46-1.29	0.88 ± 0.18	0.38-1.11	0.77 ± 0.16	0.29-26.52	0.67 ± 3.59	0.21-0.82	0.51 ± 0.13
DSW	0.08-0.36	0.21 ± 0.06	0.06-0.27	0.16 ± 0.04	0.04-0.31	0.12 ± 0.05	0.01-0.16	0.07 ± 0.03
DRW	0.08-0.30	0.17 ± 0.05	0.06-0.23	0.13 ± 0.04	0.04-0.36	0.08 ± 0.03	0.01-0.07	0.04 ± 0.01

**Table 4:** The broad sense heritability of the studied traits varied from low to high among the safflower genotypes.

Trait	MS_G	MS_G×T	MS_e	H² (broad sense)
BY	10.1174	9.5792	9.6831	0.2
DRW	0.00794	0.00257	0.0007	0.815
DSW	0.01722	0.00256	0.00155	0.772
FRW	0.0426	0.00177	0.00017	0.958
FSW	1.87637	1.86425	1.84033	0.17
NL	2.7252	0.5738	0.0035	0.948
PH	7.823	0.571	0.03	0.978
RL	4.778	0.374	0.019	0.975

**Figure 2:**
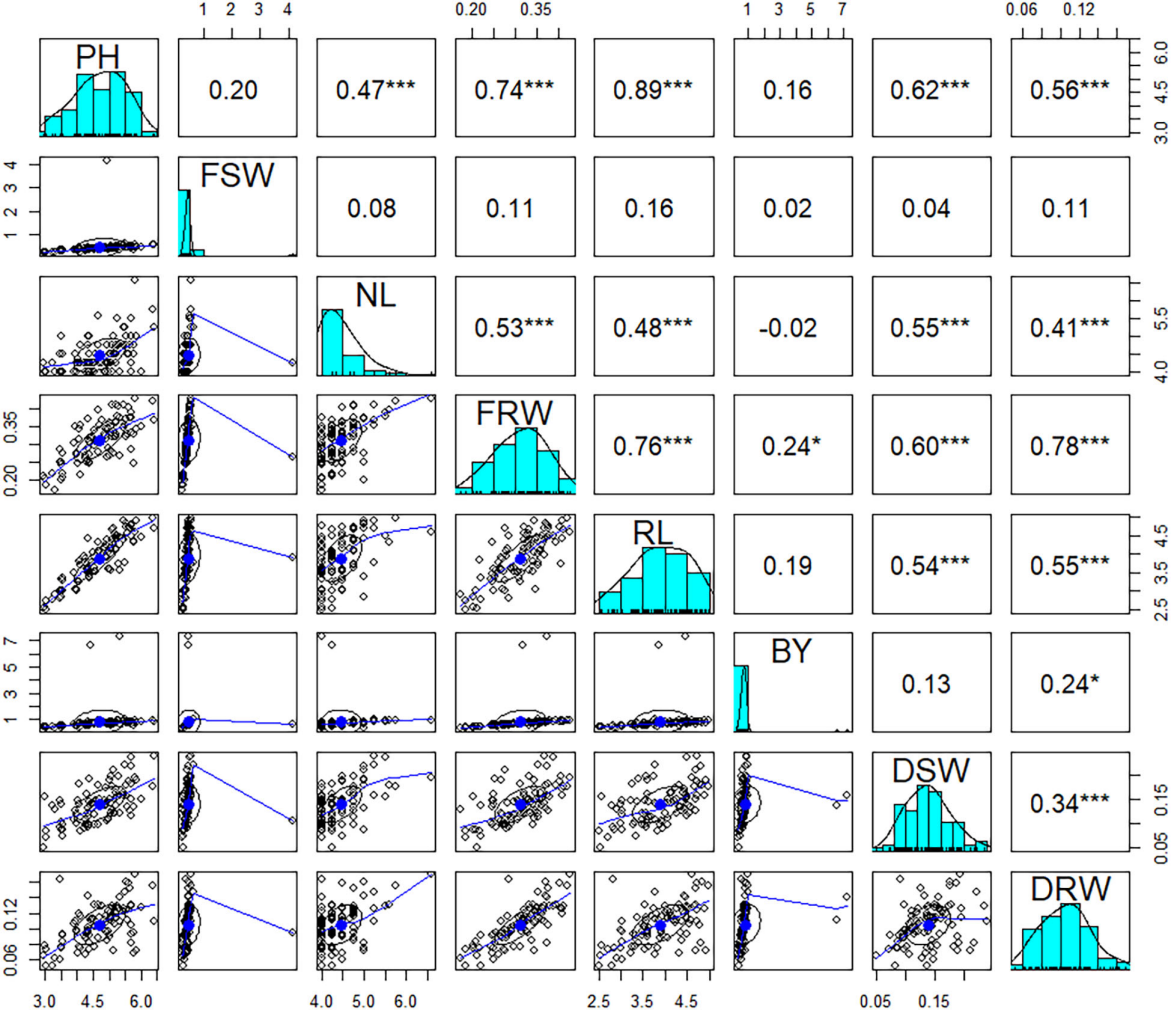
Correlation analysis among the studied traits using mean data of four treatments. BY, biological yield; DRW, dry root weight; DSW, dry shoot weight; FRW, fresh root weight; FSW, fresh shoot weight; NOL, number of leaves; PH, plant height; RL, root length. * shows significance with p < 0.05 and *** shows highly significance with p < 0.001.

The correlation analysis revealed significantly positive relationships between most of the studied traits across all treatments (**Figure 2**). In T1, all traits showed significantly positive correlations with each other. In T2, most traits exhibited significantly positive correlations, with the exception of FSW, which negatively correlated with all the studied traits. In T3, positive correlations were observed among most traits, except for NL and DRW, which negatively correlated with BY and DSW, respectively. Similarly, in T4, all traits were in positive correlation among each other (**Supplementary Table 2**). The correlation analysis using mean data across all treatments also demonstrated significantly positive correlations among all the traits examined except FSW and BY.

**Figure 3:**
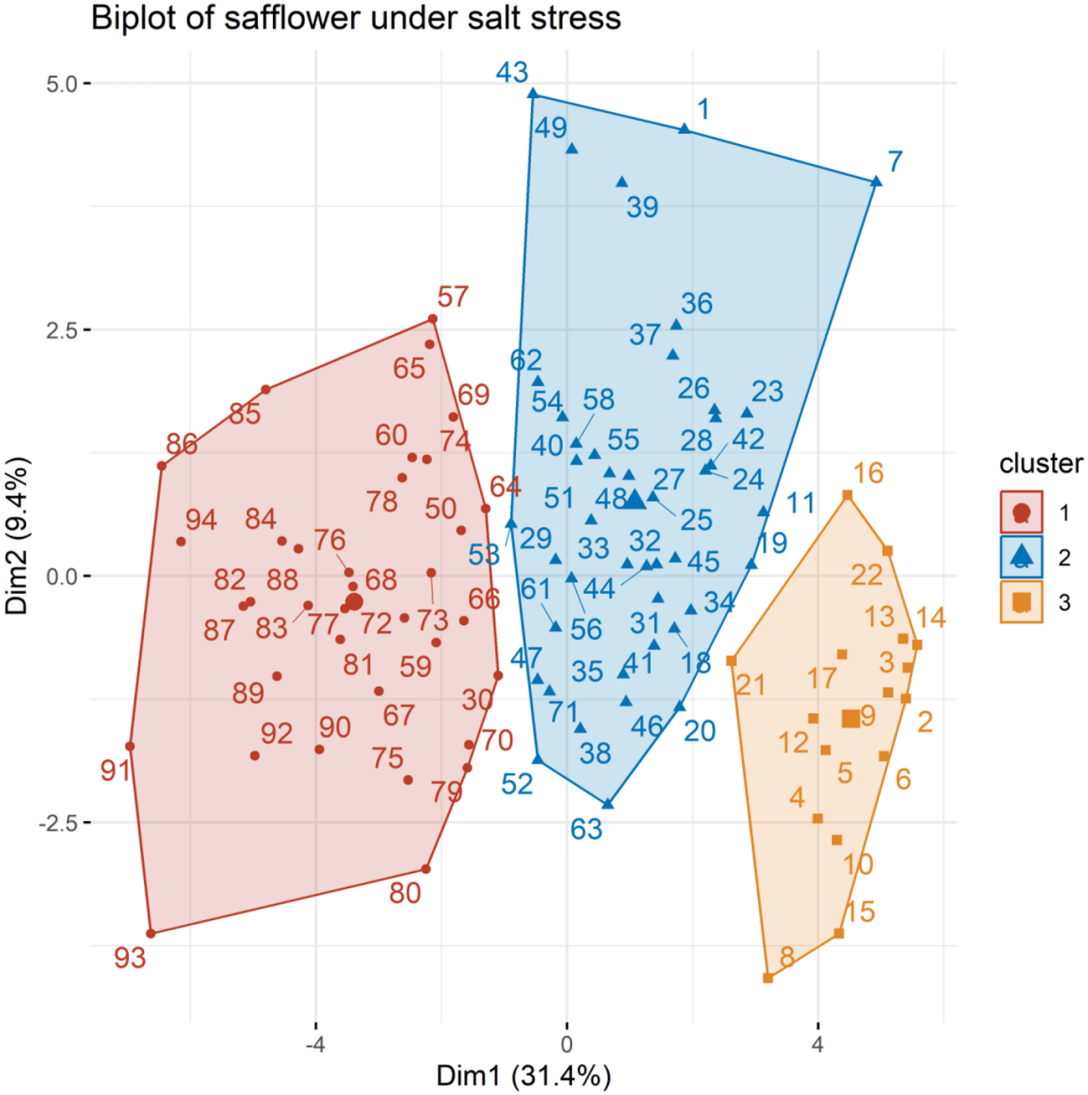
Principal component analysis separated the safflower genotypes into the three groups.

The first two principal components, each having eigenvalues of 1 or greater, explained a considerable portion of the total variation (40.8%) in PCA. The first principal component (PC1) accounted for 31.4% of the overall variance, while the second principal component (PC2) explained an additional 9.4% variance (**Figure 3**). Based on their distribution in the PCA biplot quadrants, all genotypes were categorized into three distinct groups, labeled as 1, 2, and 3.

A rigorous selection criterion identified top-performing genotypes (G-50, G-58, and G-94) from the safflower panel using genotypic data and trait mean data under all treatments (**Table 5**). The STI indicated moderate to high tolerance in the majority of genotypes across all studied traits (**Supplementary Table 3**). The genotypes ranking was further confirmed using online tool iPASTIC (**Supplementary Table 4**). Genotypes demonstrating the best mean performance and highest salt tolerance could be valuable for developing salt-tolerant cultivars.

**Table 5:** List of the best performing genotypes that could be used to develop salt tolerant safflower cultivars.

Treatment	Trait	Genotypes			Mean of best lines	% increase in the population mean	Population mean
		G-50	G-58	G-94			
Treatment 1	PH	6.80	7.63	6.47	7.05	24.96	5.64
	FSW	0.64	0.65	0.65	0.65	31.48	0.49
	NL	6.00	7.00	8.33	7.67	53.33	5.00
	FRW	0.47	0.53	0.52	0.53	32.33	0.40
	RL	5.53	5.53	5.27	5.40	16.80	4.62
	BY	1.19	1.16	1.17	1.17	32.52	0.88
	DSW	0.23	0.25	0.28	0.27	27.37	0.21
	DRW	0.20	0.25	0.26	0.25	45.39	0.17
Treatment 2	PH	5.43	6.40	5.37	5.88	18.52	4.96
	FSW	0.57	0.54	0.57	0.56	29.65	0.43
	NL	6.00	6.00	7.00	6.50	41.33	4.60
	FRW	0.42	0.44	0.47	0.46	32.29	0.35
	RL	4.67	5.40	4.47	4.93	18.78	4.15
	BY	1.02	0.99	0.99	0.99	28.82	0.77
	DSW	0.18	0.19	0.23	0.21	35.60	0.16
	DRW	0.17	0.21	0.22	0.22	69.85	0.13
Treatment 3	PH	4.53	6.03	6.03	6.03	38.29	4.36
	FSW	0.47	0.51	0.51	0.51	38.85	0.37
	NL	5.00	5.00	6.00	5.50	31.11	4.20
	FRW	0.34	0.39	0.40	0.40	38.86	0.28
	RL	4.20	4.63	4.43	4.53	24.69	3.64
	BY	0.81	0.91	0.88	0.90	38.87	0.64
	DSW	0.12	0.15	0.16	0.15	29.42	0.12
	DRW	0.09	0.11	0.13	0.12	49.69	0.08
Treatment 4	PH	4.27	5.43	5.30	5.37	40.36	3.82
	FSW	0.39	0.46	0.46	0.46	54.13	0.30
	NL	5.00	5.00	5.00	5.00	23.36	4.05
	FRW	0.29	0.32	0.32	0.32	48.80	0.22
	RL	3.53	4.37	4.27	4.32	37.07	3.15
	BY	0.71	0.82	0.74	0.78	51.07	0.51
	DSW	0.09	0.11	0.10	0.11	53.16	0.07
	DRW	0.04	0.05	0.07	0.06	56.82	0.04
Mean of all Treatments	PH	5.26	6.38	5.79	6.08	29.49	4.70
	FSW	0.52	0.54	0.55	0.54	36.78	0.40
	NL	5.50	5.75	6.58	6.17	38.21	4.46
	FRW	0.38	0.42	0.43	0.43	36.67	0.31
	RL	4.48	4.98	4.61	4.80	23.27	3.89
	BY	0.93	0.97	0.95	0.96	38.03	0.69
	DSW	0.16	0.18	0.19	0.18	33.37	0.14
	DRW	0.13	0.16	0.17	0.16	54.72	0.11

### 3.3 Marker-trait association through GWAS

**Figure 4:**
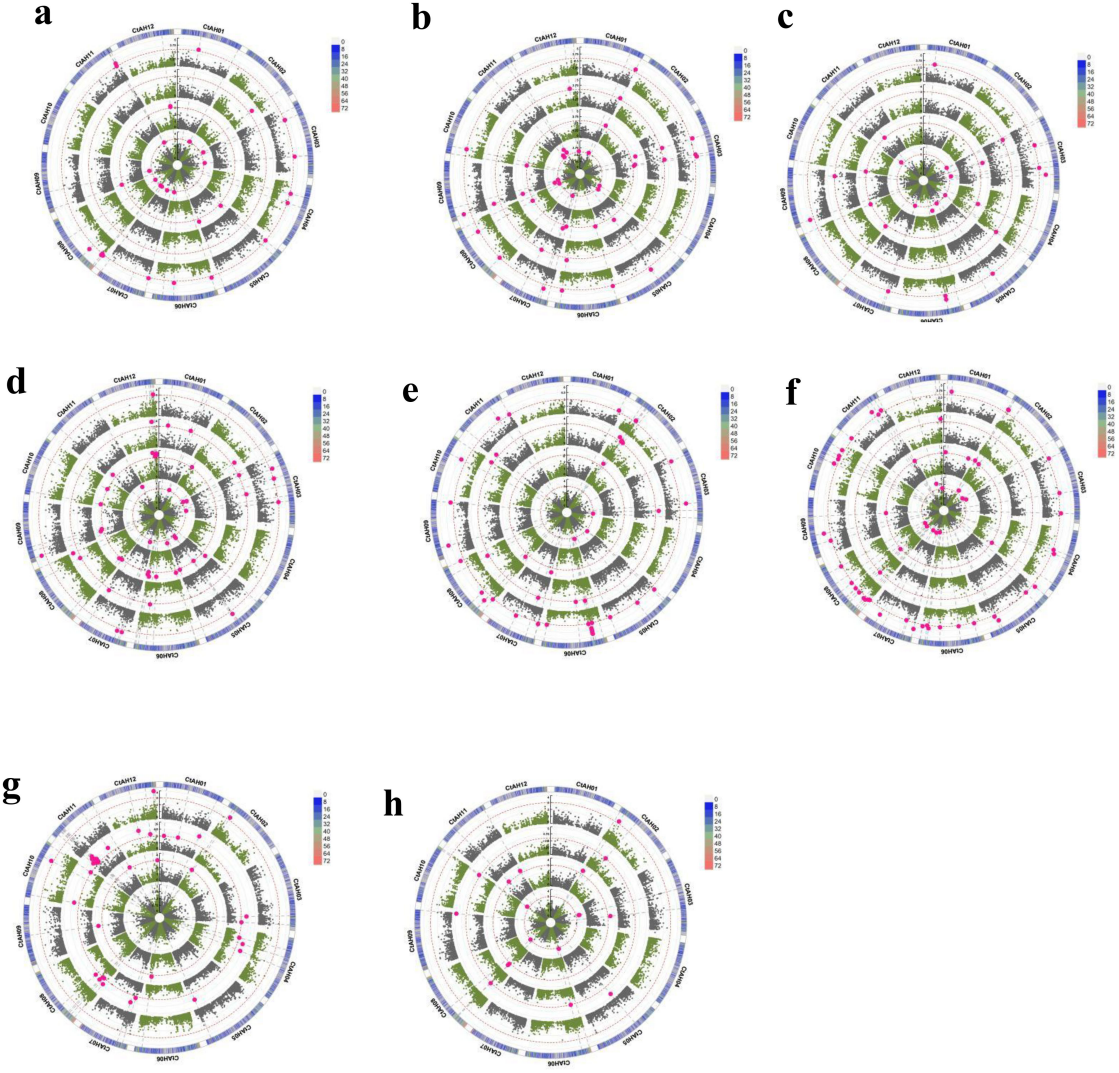
Circular pseudo-manhattan plot of **(a)** BY, **(b)** DRW, **(c)** DSW, **(d)** FRW, **(e)** FSW, **(f)** NL, **(g)** PH, **(h)** RL under T1 (inner circle), T2 (second circle), T3 (third circle) and T4 (fourth circle). The thin dotted red line indicates significance at *p*< 0.001 (−log10 = 3 or more) beyond which an association is counted as a true association (highlighted red dots). The scale between chromosomes 1 and 12 indicates the LOD threshold. The colored boxes outside on the top right side indicate the SNP density across the genome where grey to pink indicates less to denser.

**Figure 5:**
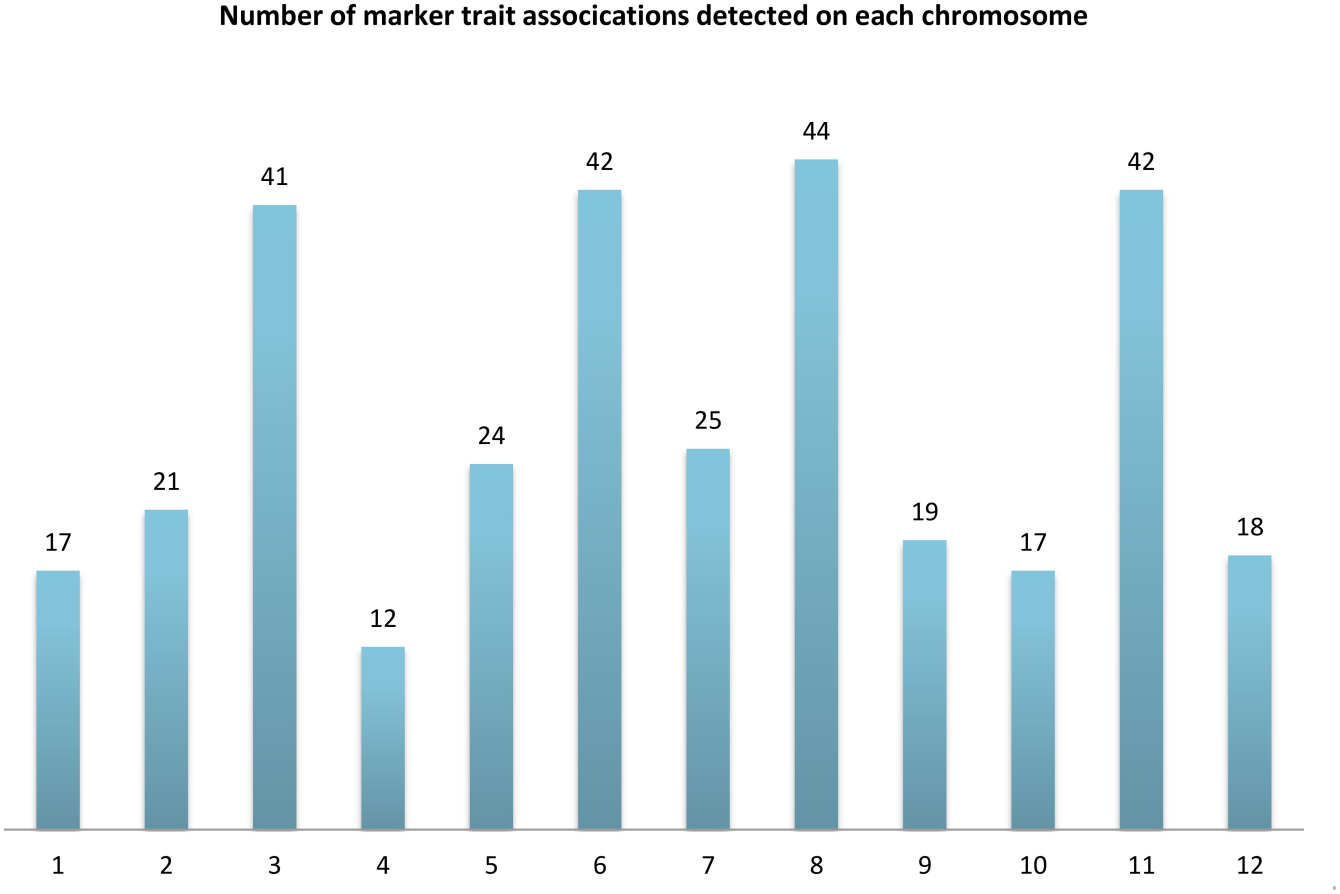
Distribution of marker-trait associations (MTAs) across 12 safflower chromosomes. Bars represent the total number of significant MTAs detected on each chromosome, ranging from 12 to 44.

In this study, 12,232 highly informative DArTseq markers - filtered from GBS data generated by Diversity Arrays Technology (DArT) for 94 safflower genotypes - were used to perform GWAS on eight morphological traits at the seedling stage using the MLM (Q + K) model. Phenotypic data from these traits, collected under four NaCl treatments under control conditions, enabled identification of environmentally stable and treatment-specific MTAs. The SNP-GWAS analysis identified 322 significant MTA loci across the eight morphological traits under all four treatments. Specifically, 34, 25, 44, and 48 MTAs were detected for BY, DRW, DSW, and FRW, respectively, while FSW, NL, PH, and RL showed 46, 60, 47, and 18 MTAs (**Supplementary Table 5**; **Figure 4**). A total of 17, 21, 41, 12, 24, and 42 MTAs were detected on chromosomes 1–6, while chromosomes 7–12 harbored 25, 44, 19, 17, 42, and 18 MTAs, respectively (**Figure 5**). Overall, these MTAs hold promise for improving morphological traits in safflower.

### 3.4 GWAS-derived genes, protein-protein interaction networks, and GO enrichment

**Figure 6:**
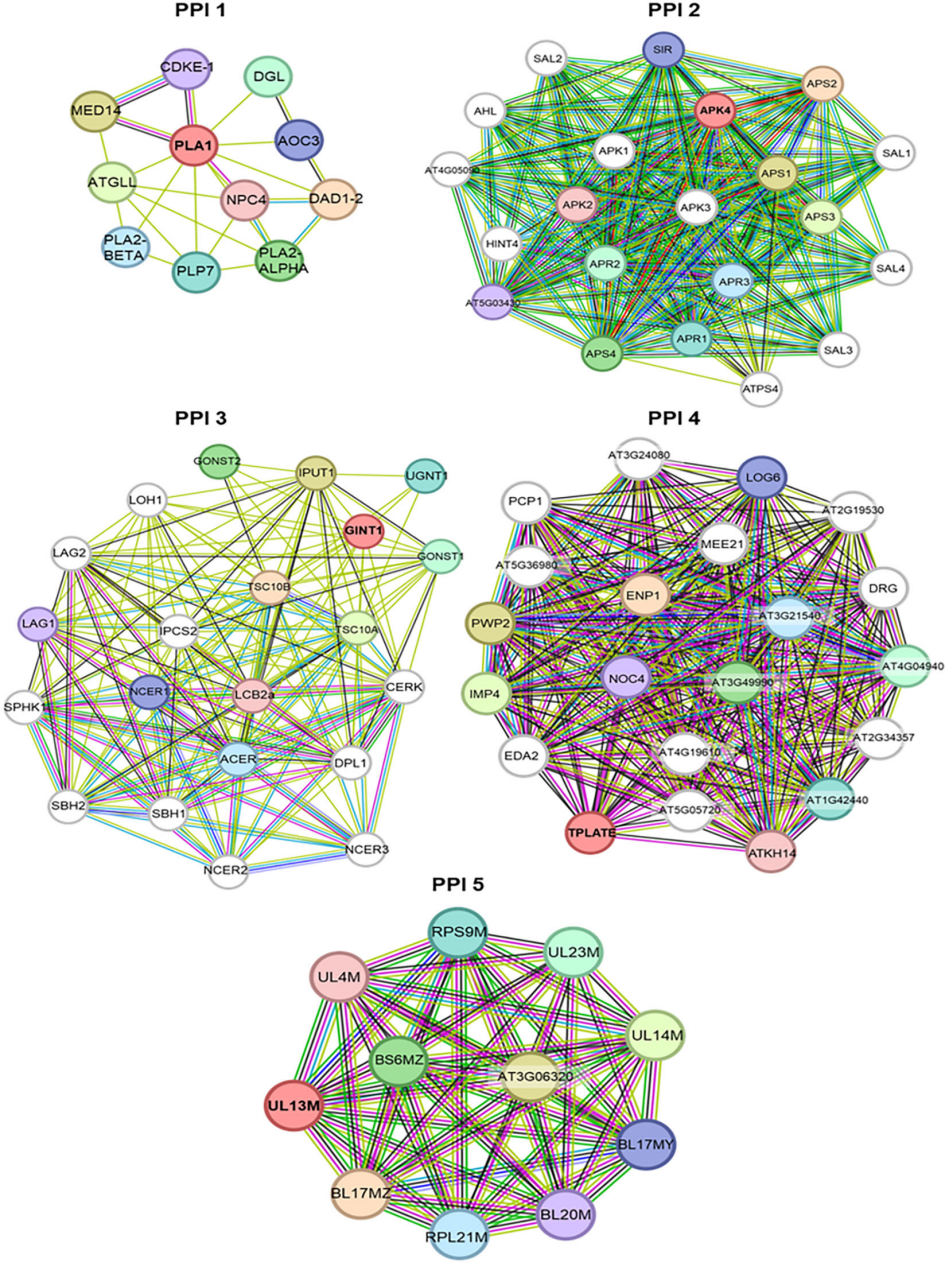
Protein–protein interaction network analysis of the loci associated with the SNP markers identified in treatment T1. Where T1 contain 0 mM concentration of NaCl. PPI networks were generated in String.

**Figure 7:**
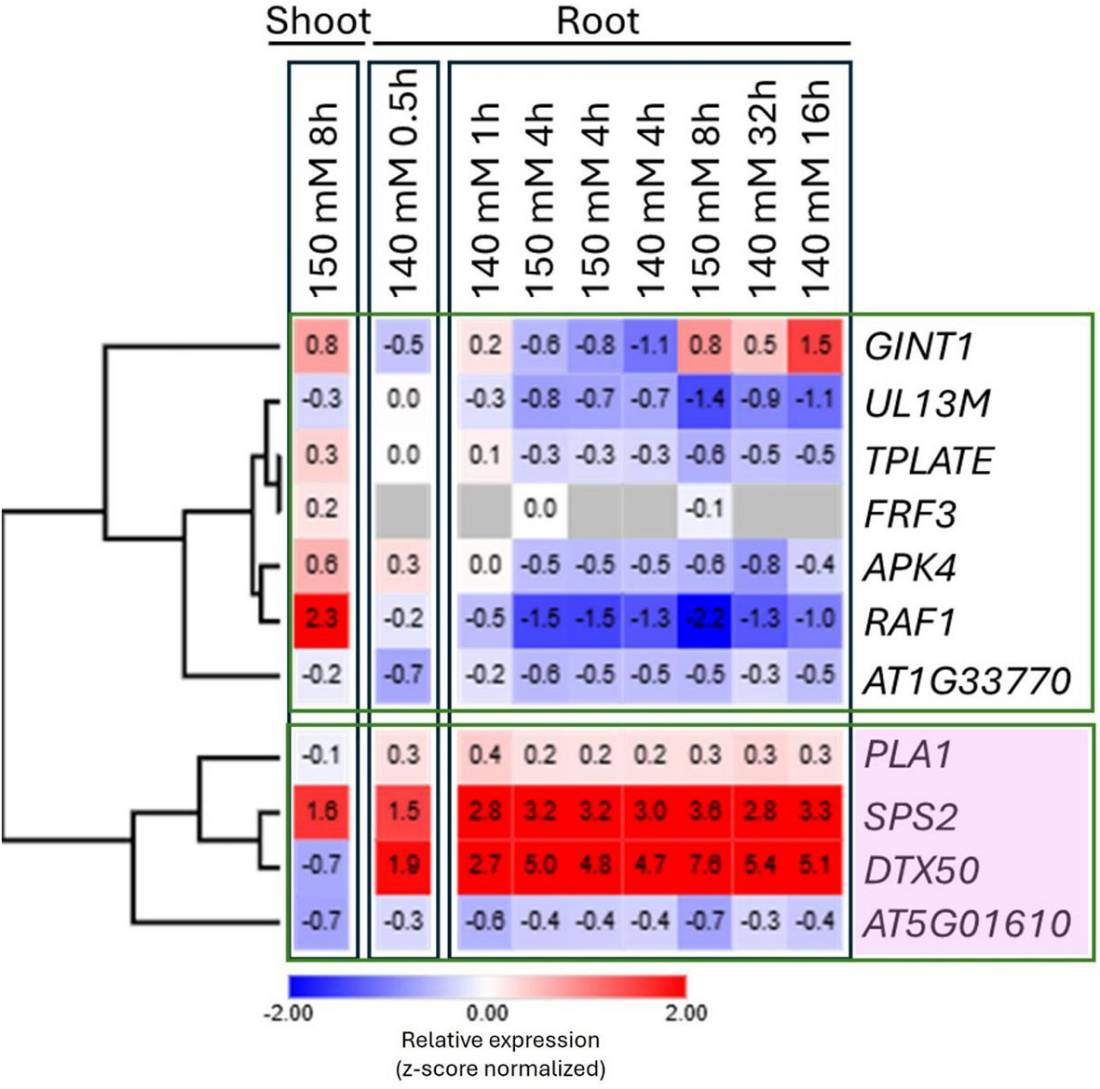
*In silico* expression profiles of *Arabidopsis* orthologs of the marker genes under salinity. Datasets GSE71001, GSE7642, GSE229217 and GSE270516 were analyzed and the heat map was generated by ggplot2 in R following Euclidean distance hierarchical clustering.

**Figure 8:**
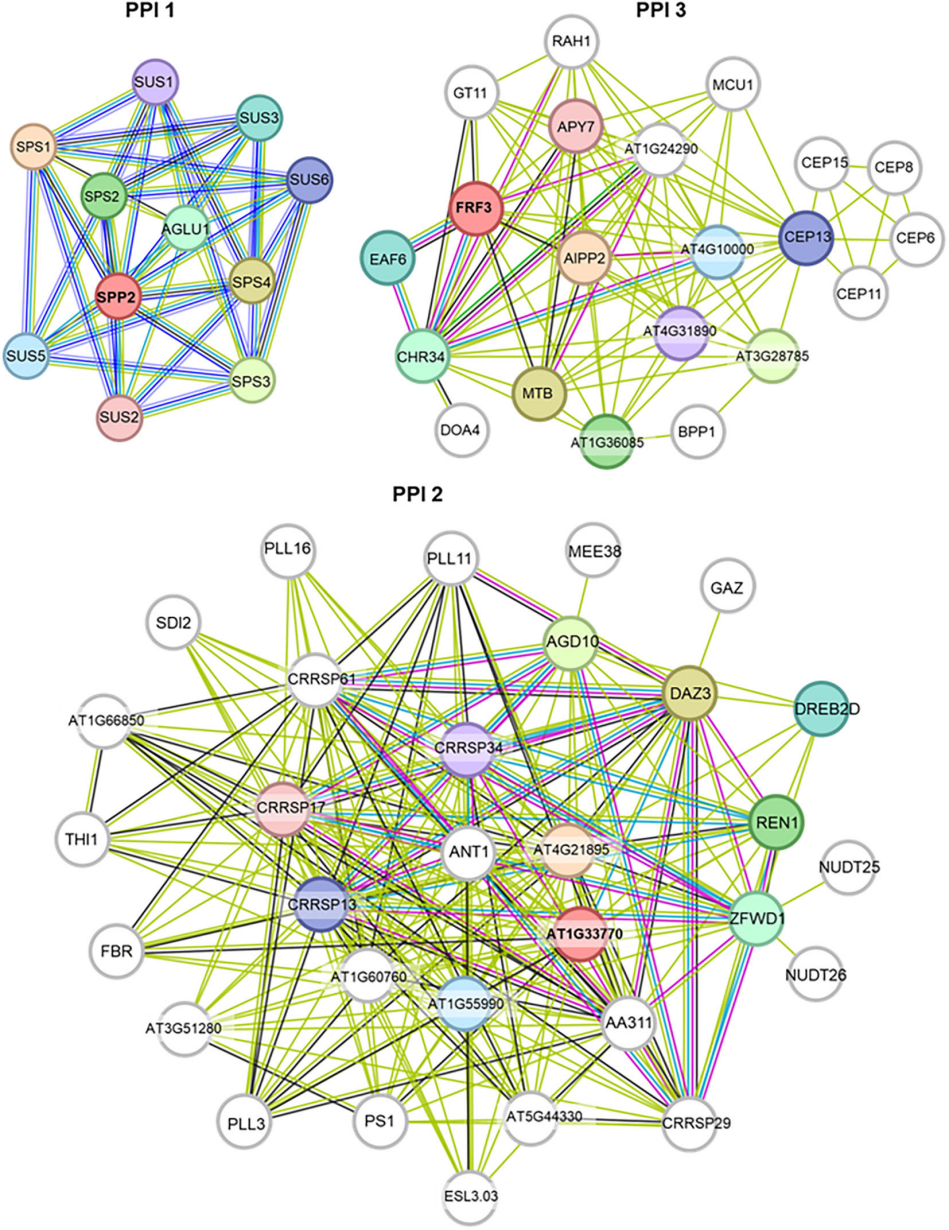
Protein–protein interaction network analysis of the loci associated with the SNP markers identified in treatment T2. Where T2 contain 80 mM concentration of NaCl. PPI networks were generated in String.

**Figure 9:**
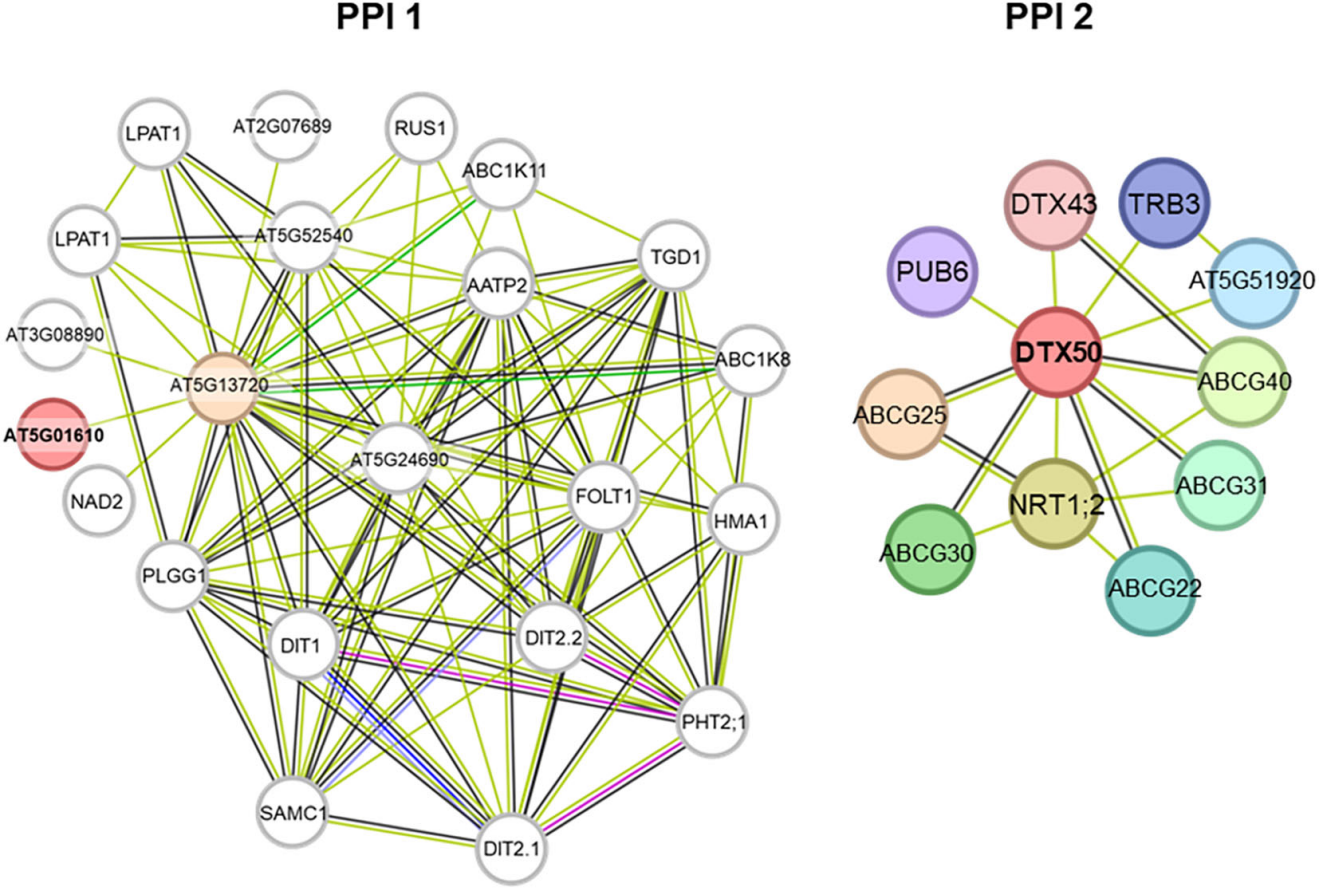
Protein–protein interaction network analysis of the loci associated with the SNP markers identified in treatment T3. Where T3 contain 160 mM concentration of NaCl. PPI networks were generated in String.

To investigate molecular mechanisms of salinity tolerance in safflower, we identified loci associated with GWAS markers and their Arabidopsis orthologues via BLAST searches (**Table 6**). Marker *DArT*-100005876 (linked to BY in T1) yielded six orthologues, while others corresponded to single orthologues. PPI networks were constructed for each orthologue across treatments (**Figures 6**–**9**; **Supplementary Table 6**). In T1, PHOSPHOLIPASE A I (PPI 1 of marker *DArT*-100005876) interacted with 11 proteins, ADENYLYL-SULFATE KINASE 4 (PPI 2) with 21, GLUCOSAMINE INOSITOLPHOSPHORYLCERAMIDE TRANSFERASE 1 (PPI 3) and TPLATE (PPI 4) each with 21, and RIBOSOMAL PROTEIN UL13M (PPI 5) with 11. In T2, networks centered on SUCROSE-PHOSPHATE SYNTHASE 2 (PPI 1, marker *DArT*-38084188; FRW; 11 proteins), AT1G33770 protein kinase (PPI 2, marker *DArT*-38083288; FRW; 31 proteins), and FAR1-RELATED SEQUENCE 3 (PPI 3, marker *DArT-45483426*; RL; 21 proteins). In T3, AT5G01610 (PPI 1, marker *DArT-*15670616; DSW; 21 proteins) and DETOXIFICATION 50 (PPI 2, marker *DArT*-100020623; DSW; 11 proteins). In T4, RALFL1 (marker *DArT*-22763645 associated with DSW) included 21 proteins. These PPI networks provide critical insights into the potential molecular interactions contributing to salinity tolerance in safflower, highlighting key candidate genes and their associated pathways.

**Table 6:** Arabidopsis orthologs of the genes.

AGI	Gene abbreviation	Gene name	Sequence similarity (%)	E value
Treatment: T1 - Trait: BY – Marker: *DArT-*100005876				
AT5G04500	*GINT1*	*GLUCOSAMINE INOSITOLPHOSPHORYLCERAMIDE TRANSFERASE 1*	41.0	3e-04
AT5G67520	*APK4*	*ADENOSINE-5’-PHOSPHOSULFATE (APS) KINASE 4*	40.1	0.001
AT3G01790	*UL13M*	*RIBOSOMAL PROTEIN UL13M*	40.1	0.001
AT3G01780	*TPLATE*	*TPLATE*	40.1	0.001
AT1G61850	*PLA1*	*PHOSPHOLIPASE A I*	40.1	0.001
AT3G22520	–	Spindle assembly abnormal protein	39.2	0.001
Treatment: T2 - Trait: FRW – Marker: *DArT-*38084188				
AT3G52340	*SPP2*	*SUCROSE-PHOSPHATASE 2*	71.6	1e-11
Treatment: T2 - Trait: FRW – Marker: *DArT-*38083288				
AT1G33770	–	Protein kinase superfamily protein	39.2	0.001
Treatment: T2 - Trait: RL – Marker: *DArT-*45483426				
AT4G12850	*FRF3*	*FAR1-RELATED SEQUENCES-RELATED FACTOR3*	39.2	0.001
Treatment: T3 - Trait: DSW – Marker: *DArT-*15670616				
AT5G01610	–	Hypothetical protein	40.1	0.001
Treatment: T3 - Trait: PH – Marker: *DArT-*100020623				
AT5G52050	*DTX50*	*DETOXIFICATION EFFLUX CARRIER 50*	41.0	3e-04
Treatment: T4 - Trait: DSW – Marker: *DArT-*22763645				
AT1G02900	*RALF1*	*RAPID ALKALINIZATION FACTOR 1*	40.1	0.001

To gain a deeper understanding of the functional relevance of the protein clusters identified in the PPI network analysis, we conducted GO enrichment analysis based on their biological functions and metabolic processes (**Supplementary Figures 1–4**). GO enrichment of PPI clusters revealed treatment-specific pathways: T1 featured lipid metabolism/jasmonic acid biosynthesis (PPI 1), sulfate assimilation and metabolism (PPI 2), sphingolipid biosynthesis (PPI 3), ribosome biogenesis and rRNA processing (PPI 4), and translation (PPI 5) (**Supplementary Figure 1**). T2 showed sucrose metabolism (PPI 1), pectin catabolism (PPI 2), and nitrate import/root regulation (PPI 3) (**Supplementary Figure 2**). T3 involved ammonia assimilation and carboxylic acid transport (PPI 1) and ABA transport (PPI 2) (**Supplementary Figure 3**). T4 highlighted protein autophosphorylation/brassinosteroid responses (**Supplementary Figure 4**). These networks illuminate interconnected pathways in safflower salinity responses.

### 3.5 Integration of GWAS and transcriptome analysis

Integrating GWAS signals with transcriptome data enhances candidate gene prioritization for salinity tolerance. Co-localization identified 11 highly expressed DEGs across eight genomic regions, categorized by z-score normalized expression into two clusters (four and seven genes; **Figure 7**). Notable upregulated genes under salt stress included *PLA1* (vacuole biogenesis/generative organ development), *SPS2* (sucrose accumulation in response to stresses like salinity, supporting metabolism), and *DTX50* (transmembrane protein in SOS pathway essential for salt stress). This convergence strengthens their candidacy for functional validation in safflower breeding.

## 4 Discussion

Breeding for salt tolerance in safflower presents a significant challenge due to the polygenic nature of the traits. Currently, limited information is available on genomic regions associated with salt tolerance at the seedling stage in safflower. To address this, we evaluated a safflower germplasm panel comprising of 94 genotypes and utilized it to identify candidate genotypes and genes responsible for salinity tolerance at the seedling stage.

### 4.1 Phenotypic variation and salt tolerant genotypes

ANOVA revealed significant differences among genotypes, treatments, and their interaction (G * T) for most studied traits, with the exceptions of BY and FSW (**Table 2**). The presence of significant differences among genotypes for the studied traits highlights the availability of genetic variation for safflower improvement, which corroborates previous findings (Ali et al., 2025). Among the treatments, T2 (80 mM) was identified as the salt dose causing the least reduction in morphological traits, while T4 showed the maximum decrease across all studied traits.

The germplasm’s overall performance in this study was significantly impacted by varying NaCl treatments (**Supplementary Figure 5**). For instance, PH was decreasing 12.06%-32.27% in T2-T4, compared with T1. FSW was reducing 14.29%-38.78% in T2-T4, compared with T1. NL was diminishing 8.00%-19.00% in T2-T4, compared with T1. FRW was declining 12.50%-45.00% in T2-T4, compared with T1. RL was decreasing 10.17%-31.82% in T2-T4, compared with T1. BY was reducing 12.50%-42.05% in T2-T4, compared with T1. DSW was diminishing 23.81%-66.67% in T2-T4, compared with T1. DRW was decreasing 23.53%-76.47% in T2-T4, compared with T1. These findings align with previous research on safflower and other crops under salt stress conditions (Gengmao et al., 2015; Uzair et al., 2022). The results also indicate that safflower exhibits moderate salt tolerance, highlighting the need for further research to identify genotypes and genes that could contribute to the development of salt-tolerant cultivars.

Previously, moderate salt tolerance capability in safflower, with 50% reduction in biomass at 125 mM NaCl (12.5 dS/m) has been reported. Growth is severely impacted at higher NaCl concentrations of 250 mM (25 dS/m) and 350 mM (35 dS/m) (Gengmao et al., 2015; Singh et al., 2013; Kaya et al., 2003, Kaya et al., 2019; Ghazizade et al., 2012). In another study on safflower, genotypes were evaluated for salt tolerance using 150 mM NaCl at the vegetative stage, with total plant biomass and photosynthetic attributes serving as selection criteria (Siddiqi et al., 2009). Genotypic variation is more pronounced under higher NaCl treatments, making moderate to severe stress conditions optimal for identifying stress tolerance in diverse germplasm.

The present study reported high heritability for most of the traits examined, except for BY and FSW, which showed low heritability estimates. Heritability values are important indicators of the potential effectiveness of selection in plant breeding. A high heritability value suggests that most of the observed variation is due to genetic differences among the plants, making selection more efficient. In contrast, low heritability indicates that the variation is largely attributable to environmental factors rather than genetics, meaning that the genotype is heavily influenced by the testing environment (Limbongan et al., 2024).

Correlation analysis reveals valuable relationships between traits, aiding crop improvement (Ali et al., 2020b). Under salt stress, significantly positive correlations exist between shoot length and root length, as well as between seedling fresh weight and both root and shoot lengths. Additionally, root fresh weight correlates positively with seedling length, root length, and seedling fresh weight (Kayaçetin, 2020; Gürsoy, 2023). These findings collectively reinforce the interconnectedness of plant growth parameters in response to salt stress. Furthermore, traits that show significant associations with one another may be useful for selecting promising genotypes.

PCA simplifies complex data into manageable groups, revealing underlying relationships. It helps select optimal genotypes by considering multiple traits (Ali et al., 2024a). In our study, the first two principal components explained 40.8% of the variation. Genotypes were grouped into three clusters based on traits like PH, FSW, RL, and BY (**Supplementary Table 7**). Group 3 had the highest PH (4.94 cm) and RL (4.08 cm), while Group 1 had the highest FSW (0.53 g) and BY (0.92 g). The best-performing genotypes identified were G-50 and G-94 from Group 1, and G-58 from Group 2.

Phenotypic data along with population mean and percent increase were employed to identify superior genotypes. Three genotypes, G-50, G-58, and G-94 were emerged as the most favorable ideotypes, demonstrating positive genetic improvements for all traits (**Table 5**). These genotypes, possessing more favorable alleles, can be utilized in safflower breeding programs to enhance traits associated with salt stress tolerance.

The STI calculation revealed that a majority of genotypes exhibited moderate to high tolerance against salt stress across all studied traits, corroborating previous findings. According to Rahman et al., 2017, higher STI values during the early seedling stage indicate greater tolerance to abiotic stress. Furthermore, the best performing safflower genotypes (G-50, G-58, and G-94) observed high tolerance (> than 0.65) against salt stress in all treatments (**Supplementary Table 3**) (Tao et al., 2021). The same genotypes were also ranked as tolerant using the iPASTIC online tool (**Supplementary Table 4**). It is therefore suggested to use the identified genotypes as parental lines for the development of salt-tolerant safflower cultivars.

### 4.2 Marker-assisted selection breeding for salt tolerance in safflower

The identification of loci influencing key plant traits is crucial for marker-assisted breeding aimed at enhancing crop productivity under changing climate conditions. Despite its importance, there are few reports identifying markers or loci associated with agronomic traits in safflower (Ali et al., 2024). Moreover, even fewer studies have reported MTAs for safflower traits under salinity stress. The number of significantly associated markers increased across treatments, with 18 markers showing associations with multiple traits or demonstrating consistency under varying salt stress conditions. Among these consistent markers, DSW was associated with 14 markers, FSW was associated with 11 markers, and DRW was associated with eight markers. Additionally, FSW was associated with three markers. Given these findings, it is recommended that the identified markers be validated and converted into Kompetitive Allele Specific PCR (KASP) assays to accelerate safflower breeding under saline stress conditions. Quantitative traits are influenced by both genetic and environmental factors, although specific details remain to be fully elucidated (Yang et al., 2021). Similar findings have been reported in crops like wheat, where 11 stable markers were identified for breeding under stress conditions (Safdar et al., 2020). This suggests a strong potential for the use of safflower markers in enhancing salt tolerance via marker-assisted breeding.

### 4.3 Candidate genes for salinity tolerance in safflower

The PPI 1 in T1 includes PLA1 enzymes, which catalyze the hydrolysis of acyl groups from phospholipids at the sn-1 position. They are crucial for generative organ development and vacuole biogenesis, including controlling polar vacuole location in zygotes for asymmetrical cell division (**Figure 6**) (Takáč et al., 2019; Kimata et al., 2019). PPI 2 in T1 included APK4, which supplies activated sulfate for sulfating secondary metabolites like glucosinolates (Halkier and Gershenzon, 2006). Sulfated metabolites contribute to plant defense against biotic/abiotic stresses, while small sulfated peptides (e.g., phytosulfokines, PSY1, RGF) regulate growth. These compounds underscore sulfation’s dual role in stress adaptation (e.g., salt tolerance) and developmental processes (**Figure 6**) (Matsuzaki et al., 2010). PPI 3 in T1 included GINT1, which mediates GIPC glycosylation by adding N-acetylglucosamine. In *Arabidopsis*, GINT1 inhibition reduces salt sensitivity, with mutants showing higher germination rates under salt stress (**Figure 6**) (Gigli-Bisceglia and Testerink, 2021). PPI 4 in T1 included TPLATE, involved in cytokinesis and localized to the cell plate. The TPLATE complex is a key adaptor for Clathrin-mediated endocytosis in plants, crucial for growth and physiological processes like nutrient uptake and pathogen defense (**Figure 6**) (Narasimhan et al., 2020). PPI 5 in T1 includes UL13M, part of the ribosomal protein L13 family. Upregulation of these proteins may enhance translation or ribosome assembly. Overexpressing a homolog in *E. coli* improved salt and freezing tolerance (**Figure 6**) (Tanaka et al., 2001).

PPI 1 in T2 includes SPP2, crucial for plant metabolism. Sucrose often accumulates in response to environmental stresses like cold, salinity, and drought (**Figure 8**) (Lunn and Ap Rees, 1990). PPI 2 involves FRF proteins from the FRS-FRF family, which regulate developmental processes and stress responses (**Figure 8**) (Jafari et al., 2025). PPI 3 includes AT1G33770, a potential protein kinase related to stress responses like salinity (**Figure 8**) (Majeed et al., 2023).

**Figure 10:**
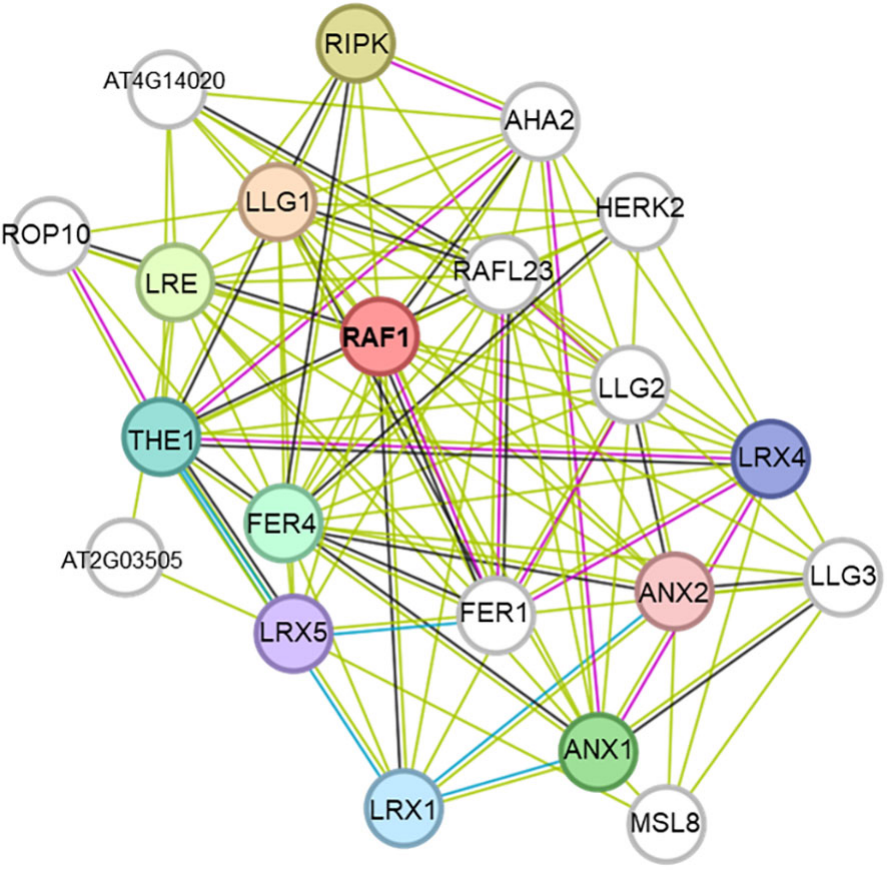
Protein–protein interaction network analysis of the loci associated with the SNP markers identified in treatment T4. Where T4 contain 250 mM concentration of NaCl. PPI networks were generated in String.

PPI 1 in T3 included AT5G01610, a hypothetical protein with a DUF538 domain, whose function remains unknown (**Figure 9**) (Ijaq et al., 2015). PPI 2 in T3 included DTX50, a transmembrane protein crucial for salt stress response via the SOS pathway, enhancing tolerance to drought, salt, and cold (**Figure 9**) (Lu et al., 2019). PPI in T4 included RAF1, which activates SnRK2 kinases for salt stress tolerance and ABA signaling (**Figure 10**) (Fàbregas et al., 2020).

Our analysis revealed that *PLA1, SPS2*, and *DTX50* formed a distinct cluster and were significantly upregulated in the roots following salinity treatment. In contrast, most of the other genes were predominantly downregulated and clustered separately. Notably, AT5G01610 was grouped with *PLA1, SPS2*, and *DTX50* despite exhibiting a slight downregulation in the roots under salinity stress. This clustering pattern suggests that *PLA1, SPS2*, and *DTX50* may play a coordinated role in the root’s adaptive response to salinity, potentially contributing to stress tolerance mechanisms (**Figure 7**). The association of AT5G01610 with this cluster, despite its mild downregulation, implies that it may share functional or regulatory interactions with these genes, warranting further investigation into its potential role in salinity adaptation.

## 5 Conclusions

We pioneered the pheno-genotypic evaluation of seedling traits at various salt concentrations in safflower providing insights into its behavior under salt stress and facilitating a comprehensive genetic analysis of MTAs with the observed traits. A concentration of 250 mM NaCl was recommended as the appropriate dose for screening salt-tolerant genotypes. Several genes potentially crucial for safflower salinity tolerance were identified. Yet, these findings require further validation through functional studies, such as gene expression analysis, overexpression or knockout experiments, to substantiate the role of specific genes in controlling safflower traits. Additionally, targeting these genes via gene editing can be important in developing salinity-tolerant safflower cultivars. Future research could benefit from expanding this to encompass a broader representation of safflower genetic diversity. We encourage safflower community to build upon these results in future studies, using them as a foundation for breeding more resilient, salt-tolerant genotypes.

## Data Availability

The datasets presented in this study can be found in online repositories. The names of the repository/repositories and accession number(s) can be found in the article/**Supplementary Material**.

## Supplementary material

Supplementary Table 1 List of safflower genotypes.

Supplementary Table 2 **(a).** Correlation analysis among the studied traits based on the mean data from Treatment 1. (**b).** Correlation analysis among the studied traits based on the mean data from Treatment 2. **(c)** Correlation analysis among the studied traits based on the mean data from Treatment 3. **(d)** Correlation analysis among the studied traits based on the mean data from Treatment 4.

Supplementary Table 3 Stress tolerance index values exhibited the salt stress tolerance potential of the tested safflower genotypes.

Supplementary Table 4 Ranking of safflower genotypes for RL in T4 based on Stress Tolerance Index (STI), calculated using the online iPASTIC tool.

Supplementary Table 5 Genome-wide association mapping identified 322 marker-trait associations using MLM model.

Supplementary Table 6 Linkage disequilibrium (LD) analysis identified markers grouped within blocks.

Supplementary Table 7 Principal component analysis revealed variability among the three groups, based on mean values for PH, FSW, RL, and BY.
