# Supplementary material for: Genome wide association studies reveal candidate genes for salt tolerance in safflower (Carthamus tinctorius L.) at seedling stage
Source: Front Plant Sci. 2026 Mar 6;17:1630492. doi: 10.3389/fpls.2026.1630492 (PMC13003225; doi:10.3389/fpls.2026.1630492)
Supplement: Supplementary Table 1 — List of safflower genotypes. [file Table1.docx]

**Supplementary Table 1.** List of safflower genotypes.

| S.No | Country Origin | Plant ID |
| --- | --- | --- |
| Genotype1 | Afghanistan | P1-253764 |
| Genotype 2 | Afghanistan | P1-304592 |
| Genotype3 | Afghanistan | PI 220647 |
| Genotype4 | Argentina | P1-367833 |
| Genotype5 | Australia | PI 235660 |
| Genotype6 | Austria | PI 253519 |
| Genotype7 | Austria | BVAL-901352 |
| Genotype8 | Bangladesh | PI-401472 |
| Genotype9 | Bangladesh | PI-401478 |
| Genotype10 | Bangladesh | PI-401480 |
| Genotype11 | Bangladesh | PI 401470 |
| Genotype12 | China | P1-262452 |
| Genotype13 | China | P1-262453 |
| Genotype14 | China | PI 543979 |
| Genotype15 | China | PI 543982 |
| Genotype16 | China | PI 544001 |
| Genotype17 | China | PI 568809 |
| Genotype18 | China | PI 568874 |
| Genotype19 | Egypt | P1-250082 |
| Genotype20 | Egypt | P1-250528 |
| Genotype21 | Egypt | P1-250532 |
| Genotype22 | Egypt | P1-250540 |
| Genotype23 | Egypt | P1-250605 |
| Genotype24 | Egypt | P1-250608 |
| Genotype25 | France | PI 576985 |
| Genotype26 | Hungary | PI 288983 |
| Genotype27 | India | P1-250601 |
| Genotype28 | India | P1-305195 |
| Genotype29 | India | P1-306926 |
| Genotype30 | India | P1-306941 |
| Genotype31 | India | P1-306976 |
| Genotype32 | India | PI 199878 |
| Genotype33 | Iran | P1-250720 |
| Genotype34 | Iran | P1-304444 |
| Genotype35 | Iran | P1-304448 |
| Genotype36 | Iran | P1-405958 |
| Genotype37 | Iran | P1-405967 |
| Genotype38 | Iran | PI 250840 |
| Genotype39 | Iran | PI 406010 |
| Genotype40 | Israel | P1-198990 |
| Genotype41 | Israel | P1-253386 |
| Genotype42 | Israel | P1-253892 |
| Genotype43 | Israel | PI 251290 |
| Genotype44 | Iraq | P1-253761 |
| Genotype45 | Iraq | P1-253762 |
| Genotype46 | Jordan | P1-251284 |
| Genotype47 | Jordan | P1-251285 |
| Genotype48 | Jordan | PI 251265 |
| Genotype49 | Jordan | PI 251267 |
| Genotype50 | Jordan | PI 251268 |
| Genotype51 | Kazakhstan | P1-314650 |
| Genotype52 | Libya | PI 393499 |
| Genotype53 | Morocco | P1-239042 |
| Genotype54 | Morocco | P1-253560 |
| Genotype55 | Pakistan | P1-250194 |
| Genotype56 | Pakistan | P1-250201 |
| Genotype57 | Pakistan | P1-250345 |
| Genotype58 | Pakistan | P1-250346 |
| Genotype59 | Pakistan | P1-250351 |
| Genotype60 | Pakistan | P1-250353 |
| Genotype61 | Pakistan | P1-250481 |
| Genotype62 | Pakistan | PI 250474 |
| Genotype63 | Pakistan | PI 250478 |
| Genotype64 | Pakistan | PI 426521 |
| Genotype65 | Pakistan | Thori-78 |
| Genotype66 | Portugal | P1-253553 |
| Genotype67 | Portugal | P1-253556 |
| Genotype68 | Portugal | P1-253564 |
| Genotype69 | Portugal | P1-253569 |
| Genotype70 | Portugal | P1-253571 |
| Genotype71 | Portugal | P1-258412 |
| Genotype72 | Romania | P1-209287 |
| Genotype73 | Russia | P1-305535 |
| Genotype74 | Spain | P1-253388 |
| Genotype75 | Spain | P1-253391 |
| Genotype76 | Spain | P1-253394 |
| Genotype77 | Spain | P1-253395 |
| Genotype78 | Syria | P1-253898 |
| Genotype79 | Syria | P1-253900 |
| Genotype80 | Syria | P1-386174 |
| Genotype81 | Thailand | P1-387821 |
| Genotype82 | Turkey | P1-304498 |
| Genotype83 | Turkey | P1-304502 |
| Genotype84 | Turkey | P1-304504 |
| Genotype85 | Turkey | P1-304505 |
| Genotype86 | Turkey | P1-340086 |
| Genotype87 | Turkey | PI 237538 |
| Genotype88 | Turkey | PI 251978 |
| Genotype89 | Turkey | PI 251984 |
| Genotype90 | Turkey | PI 406701 |
| Genotype91 | Turkey | PI 406702 |
| Genotype92 | Uzbekistan | P1-262435 |
| Genotype93 | Uzbekistan | P1-369846 |
| Genotype94 | Uzbekistan | P1-369853 |
