# Supplementary material for: Genome wide association studies reveal candidate genes for salt tolerance in safflower (Carthamus tinctorius L.) at seedling stage
Source: Front Plant Sci. 2026 Mar 6;17:1630492. doi: 10.3389/fpls.2026.1630492 (PMC13003225; doi:10.3389/fpls.2026.1630492)
Supplement: Supplementary Table 2 — (a). Correlation analysis among the studied traits based on the mean data from Treatment 1. (b). Correlation analysis among the studied traits based on the mean data from Treatment 2. (c) Correlation analysis among the studied traits based on the mean data from Treatment 3. (d) Correlation analysis among the studied traits based on the mean data from Treatment 4. [file Table2.docx]

**Supplementary Table 2a.** Correlation analysis among the studied traits based on the mean data from Treatment 1.

|  | BY | DRW | DSW | FRW | FSW | NL | PH |
| --- | --- | --- | --- | --- | --- | --- | --- |
| DRW | 0.8477^****^ |  |  |  |  |  |  |
| DSW | 0.6543^****^ | 0.5635^****^ |  |  |  |  |  |
| FRW | 0.9260^****^ | 0.8876^****^ | 0.6578^****^ |  |  |  |  |
| FSW | 0.9248^****^ | 0.7862^****^ | 0.7073^****^ | 0.8680^****^ |  |  |  |
| NL | 0.5675^****^ | 0.5028^****^ | 0.5105^****^ | 0.5194^****^ | 0.5801^****^ |  |  |
| PH | 0.7935^****^ | 0.6757^****^ | 0.7025^****^ | 0.7598^****^ | 0.8296^****^ | 0.4744^****^ |  |
| RL | 0.7482^****^ | 0.6473^****^ | 0.6265^****^ | 0.7359^****^ | 0.7415^****^ | 0.4536^****^ | 0.8890^****^ |

p < 0.0001: **** indicating a very high level of statistical significance.

**Supplementary Table 2b.** Correlation analysis among the studied traits based on the mean data from Treatment 2.

|  | By | DRW | DSW | FRW | FSW | NL | PH |
| --- | --- | --- | --- | --- | --- | --- | --- |
| DRW | 0.6813^****^ |  |  |  |  |  |  |
| DSW | 0.6750^****^ | 0.2948^**^ |  |  |  |  |  |
| FRW | 0.9176^****^ | 0.7123^****^ | 0.6218^****^ |  |  |  |  |
| FSW | 0.0171^ns^ | 0.0225^ns^ | -0.0428^ns^ | 0.0085^ns^ |  |  |  |
| NL | 0.4372^****^ | 0.2785^**^ | 0.5046^****^ | 0.4363^****^ | -0.0612^ns^ |  |  |
| PH | 0.7331^****^ | 0.4161^****^ | 0.6198^****^ | 0.6640^****^ | 0.0809^ns^ | 0.2901^**^ |  |
| RL | 0.7630^****^ | 0.4565^****^ | 0.5681^****^ | 0.7073^****^ | 0.0563^ns^ | 0.2965^**^ | 0.8447^****^ |

p < 0.01: ** indicating statistical significance at the 1% level. p < 0.0001: **** indicating a very high level of statistical significance. ns: non-significant.

**Supplementary Table 2c.** Correlation analysis among the studied traits based on the mean data from Treatment 3.

|  | By | DRW | DSW | FRW | FSW | NL | PH |
| --- | --- | --- | --- | --- | --- | --- | --- |
| DRW | 0.036^ns^ |  |  |  |  |  |  |
| DSW | 0.0334^ns^ | -0.0905^ns^ |  |  |  |  |  |
| FRW | 0.1277^ns^ | 0.2306^ns^ | 0.2379* |  |  |  |  |
| FSW | 0.0916^ns^ | 0.2126* | 0.3167 ^**^ | 0.8492**** |  |  |  |
| NL | -0.054^ns^ | 0.0336^ns^ | 0.2372* | 0.3795*** | 0.3949*** |  |  |
| PH | 0.0518^ns^ | 0.0912^ns^ | 0.1448^ns^ | 0.6248**** | 0.6777**** | 0.2942** |  |
| RL | 0.0315^ns^ | 0.0217^ns^ | 0.0948^ns^ | 0.6588**** | 0.6533**** | 0.3245** | 0.7726**** |

p < 0.05: * indicating statistical significance at the 5% level. p < 0.01: ** indicating statistical significance at the 1% level. p < 0.001: *** indicating statistical significance at the 0.1% level. p < 0.0001: **** indicating a very high level of statistical significance. ns: non-significant.

**Supplementary Table 2d.** Correlation analysis among the studied traits based on the mean data from Treatment 4.

|  | By | DRW | DSW | FRW | FSW | NL | PH |
| --- | --- | --- | --- | --- | --- | --- | --- |
| DRW | 0.3612*** |  |  |  |  |  |  |
| DSW | 0.4474**** | 0.1879* |  |  |  |  |  |
| FRW | 0.905**** | 0.3012** | 0.4658**** |  |  |  |  |
| FSW | 0.8957**** | 0.3205** | 0.5116**** | 0.872**** |  |  |  |
| NL | 0.2988** | 0.2426* | 0.2504* | 0.2698** | 0.2767** |  |  |
| PH | 0.602**** | 0.3209** | 0.4993**** | 0.5895**** | 0.6998**** | 0.1403* |  |
| RL | 0.745**** | 0.2582* | 0.4422**** | 0.7077**** | 0.7514**** | 0.2994** | 0.7847**** |

p < 0.05: * indicating statistical significance at the 5% level. p < 0.01: ** indicating statistical significance at the 1% level. p < 0.001: *** indicating statistical significance at the 0.1% level. p < 0.0001: **** indicating a very high level of statistical significance. ns: non-significant.
