# Supplementary material for: Genome wide association studies reveal candidate genes for salt tolerance in safflower (Carthamus tinctorius L.) at seedling stage
Source: Front Plant Sci. 2026 Mar 6;17:1630492. doi: 10.3389/fpls.2026.1630492 (PMC13003225; doi:10.3389/fpls.2026.1630492)
Supplement: Supplementary Table 3 — Stress tolerance index values exhibited the salt stress tolerance potential of the tested safflower genotypes. [file Table3.docx]

**Supplementary Table 3.** Stress tolerance index values exhibited the salt stress tolerance potential of the tested safflower genotypes.

| Treatment | Genotype | Trait | | | | | | | |
| --- | --- | --- | --- | --- | --- | --- | --- | --- | --- |
|  |  | PH | FSW | NL | FRW | RL | BY | DSW | DRW |
| Treatment 2 | 1 | 0.77 | 0.82 | 0.80 | 0.88 | 0.79 | 0.81 | 0.60 | 0.55 |
|  | 2 | 0.62 | 0.80 | 1.00 | 0.85 | 0.75 | 0.86 | 0.72 | 0.67 |
|  | 3 | 0.76 | 0.88 | 0.86 | 0.81 | 0.72 | 0.81 | 0.72 | 0.54 |
|  | 4 | 0.89 | 0.67 | 1.00 | 0.78 | 0.86 | 0.83 | 0.75 | 0.62 |
|  | 5 | 1.00 | 0.88 | 1.00 | 0.82 | 0.94 | 0.87 | 0.87 | 0.75 |
|  | 6 | 0.94 | 0.84 | 0.87 | 0.85 | 0.97 | 0.87 | 0.75 | 0.59 |
|  | 7 | 0.93 | 0.87 | 1.00 | 0.70 | 0.85 | 0.86 | 0.78 | 0.52 |
|  | 8 | 0.90 | 0.94 | 0.83 | 0.90 | 1.00 | 0.89 | 0.77 | 0.57 |
|  | 9 | 0.98 | 0.88 | 0.83 | 0.92 | 0.98 | 0.93 | 0.61 | 0.42 |
|  | 10 | 0.96 | 0.93 | 0.83 | 0.92 | 0.92 | 0.92 | 0.71 | 0.45 |
|  | 11 | 0.98 | 0.92 | 0.80 | 0.92 | 0.97 | 0.96 | 0.77 | 0.51 |
|  | 12 | 0.84 | 0.93 | 1.00 | 0.84 | 0.95 | 0.91 | 0.79 | 0.53 |
|  | 13 | 1.11 | 0.86 | 1.00 | 0.87 | 0.88 | 0.86 | 0.70 | 0.55 |
|  | 14 | 0.76 | 0.80 | 0.83 | 0.79 | 0.79 | 0.76 | 0.72 | 0.46 |
|  | 15 | 0.85 | 0.93 | 1.00 | 0.94 | 0.87 | 0.92 | 0.87 | 0.50 |
|  | 16 | 0.87 | 0.80 | 1.00 | 0.83 | 0.82 | 0.81 | 0.83 | 0.47 |
|  | 17 | 0.88 | 0.95 | 0.83 | 0.94 | 0.82 | 0.95 | 0.71 | 0.55 |
|  | 18 | 0.89 | 0.85 | 0.80 | 0.78 | 0.93 | 0.84 | 0.62 | 0.49 |
|  | 19 | 0.88 | 0.86 | 0.83 | 0.82 | 0.99 | 0.80 | 0.77 | 0.43 |
|  | 20 | 0.98 | 0.79 | 1.00 | 0.82 | 0.83 | 0.81 | 0.68 | 0.49 |
|  | 21 | 0.81 | 0.91 | 0.83 | 0.84 | 0.85 | 0.86 | 0.74 | 0.47 |
|  | 22 | 0.97 | 0.92 | 1.00 | 0.88 | 0.97 | 0.89 | 0.65 | 0.53 |
|  | 23 | 0.96 | 0.75 | 0.80 | 0.76 | 0.82 | 0.80 | 0.73 | 0.60 |
|  | 24 | 0.95 | 0.92 | 1.00 | 0.84 | 0.98 | 0.91 | 0.80 | 0.55 |
|  | 25 | 0.95 | 0.96 | 1.00 | 0.87 | 0.91 | 0.86 | 0.62 | 0.67 |
|  | 26 | 0.98 | 0.95 | 1.25 | 0.95 | 1.00 | 0.83 | 0.84 | 0.49 |
|  | 27 | 0.98 | 0.93 | 1.20 | 0.94 | 0.85 | 0.83 | 0.67 | 0.54 |
|  | 28 | 0.80 | 0.88 | 1.00 | 0.88 | 0.95 | 0.87 | 0.77 | 0.55 |
|  | 29 | 0.95 | 0.89 | 1.00 | 0.84 | 0.95 | 0.88 | 0.73 | 0.54 |
|  | 30 | 0.98 | 0.92 | 0.80 | 0.83 | 0.91 | 0.88 | 0.76 | 0.55 |
|  | 31 | 0.93 | 0.97 | 0.83 | 0.89 | 0.85 | 0.93 | 0.83 | 0.56 |
|  | 32 | 0.96 | 0.82 | 0.83 | 0.88 | 0.95 | 0.79 | 0.54 | 0.59 |
|  | 33 | 0.97 | 0.94 | 1.00 | 0.91 | 0.85 | 0.92 | 0.71 | 0.63 |
|  | 34 | 0.99 | 0.93 | 1.00 | 0.85 | 0.91 | 0.86 | 0.78 | 0.70 |
|  | 35 | 0.86 | 0.84 | 0.80 | 0.81 | 0.82 | 0.85 | 0.72 | 0.71 |
|  | 36 | 0.84 | 0.84 | 1.00 | 0.87 | 0.95 | 0.82 | 0.80 | 0.61 |
|  | 37 | 0.95 | 0.86 | 1.00 | 0.87 | 0.87 | 0.88 | 0.76 | 0.71 |
|  | 38 | 0.89 | 0.93 | 0.80 | 0.89 | 0.99 | 0.93 | 0.72 | 0.76 |
|  | 39 | 0.82 | 0.88 | 1.00 | 0.88 | 0.84 | 0.89 | 0.67 | 0.59 |
|  | 40 | 0.94 | 0.93 | 1.00 | 0.86 | 0.97 | 0.93 | 0.62 | 0.68 |
|  | 41 | 0.92 | 0.92 | 1.00 | 0.87 | 0.95 | 0.93 | 0.81 | 0.73 |
|  | 42 | 0.89 | 0.86 | 0.80 | 0.91 | 0.89 | 0.90 | 0.79 | 0.70 |
|  | 43 | 0.85 | 0.83 | 1.00 | 0.84 | 0.93 | 0.83 | 0.76 | 0.57 |
|  | 44 | 0.88 | 0.93 | 0.80 | 0.92 | 0.86 | 0.94 | 0.77 | 0.76 |
|  | 45 | 0.85 | 0.96 | 0.83 | 0.91 | 0.92 | 0.92 | 0.68 | 0.55 |
|  | 46 | 0.94 | 0.95 | 0.80 | 0.90 | 0.94 | 0.90 | 0.76 | 0.60 |
|  | 47 | 0.93 | 0.87 | 1.00 | 0.88 | 0.90 | 0.87 | 0.77 | 0.55 |
|  | 48 | 0.90 | 0.96 | 1.00 | 0.81 | 0.94 | 0.88 | 0.80 | 0.93 |
|  | 49 | 0.88 | 0.81 | 1.00 | 0.92 | 0.93 | 0.84 | 0.80 | 0.66 |
|  | 50 | 0.80 | 0.90 | 1.00 | 0.91 | 0.84 | 0.86 | 0.78 | 0.63 |
|  | 51 | 0.90 | 0.84 | 0.80 | 0.93 | 0.90 | 0.88 | 0.69 | 0.90 |
|  | 52 | 0.80 | 0.85 | 0.80 | 0.84 | 0.85 | 0.87 | 0.76 | 0.55 |
|  | 53 | 0.82 | 0.84 | 1.00 | 0.88 | 0.89 | 0.87 | 0.71 | 0.61 |
|  | 54 | 0.97 | 0.83 | 1.00 | 0.92 | 0.86 | 0.90 | 0.80 | 0.70 |
|  | 55 | 0.75 | 0.86 | 1.00 | 0.90 | 0.87 | 0.90 | 0.78 | 0.67 |
|  | 56 | 0.94 | 0.86 | 1.00 | 0.98 | 0.88 | 0.92 | 0.69 | 0.72 |
|  | 57 | 0.85 | 0.81 | 1.00 | 0.86 | 0.87 | 0.89 | 0.78 | 0.65 |
|  | 58 | 0.84 | 0.83 | 0.86 | 0.83 | 0.98 | 0.85 | 0.77 | 0.63 |
|  | 59 | 0.82 | 0.87 | 1.00 | 0.88 | 0.95 | 0.89 | 0.66 | 0.61 |
|  | 60 | 0.89 | 0.71 | 1.00 | 0.79 | 0.84 | 0.82 | 0.72 | 0.70 |
|  | 61 | 0.93 | 0.90 | 0.80 | 0.92 | 0.95 | 0.90 | 0.82 | 0.64 |
|  | 62 | 0.83 | 0.83 | 0.80 | 0.85 | 0.95 | 0.88 | 0.74 | 0.56 |
|  | 63 | 0.83 | 0.85 | 1.00 | 0.84 | 0.87 | 0.85 | 0.65 | 0.59 |
|  | 64 | 0.95 | 0.94 | 1.00 | 0.81 | 0.86 | 0.92 | 0.74 | 0.60 |
|  | 65 | 0.72 | 0.82 | 1.00 | 0.86 | 0.84 | 0.68 | 0.78 | 0.65 |
|  | 66 | 0.90 | 0.84 | 1.00 | 0.88 | 0.97 | 0.84 | 0.80 | 0.61 |
|  | 67 | 0.85 | 0.85 | 1.00 | 0.84 | 0.86 | 0.88 | 0.80 | 0.61 |
|  | 68 | 0.81 | 0.89 | 1.00 | 0.81 | 0.84 | 0.89 | 0.74 | 0.69 |
|  | 69 | 0.82 | 0.81 | 0.80 | 0.89 | 0.93 | 0.87 | 0.79 | 0.64 |
|  | 70 | 0.81 | 0.85 | 0.80 | 0.79 | 0.94 | 0.87 | 0.75 | 0.53 |
|  | 71 | 0.92 | 0.81 | 1.00 | 0.81 | 0.89 | 0.86 | 0.78 | 0.55 |
|  | 72 | 0.93 | 0.82 | 0.83 | 0.83 | 0.94 | 0.76 | 0.76 | 0.53 |
|  | 73 | 0.87 | 0.91 | 0.80 | 0.86 | 0.83 | 0.91 | 0.74 | 0.61 |
|  | 74 | 0.83 | 0.91 | 1.00 | 0.88 | 0.80 | 0.87 | 0.82 | 0.56 |
|  | 75 | 0.91 | 0.76 | 0.83 | 0.87 | 0.91 | 0.87 | 0.71 | 0.63 |
|  | 76 | 0.81 | 0.91 | 1.00 | 0.84 | 0.86 | 0.87 | 0.72 | 0.59 |
|  | 77 | 0.84 | 0.83 | 0.88 | 0.85 | 0.89 | 0.87 | 0.80 | 0.59 |
|  | 78 | 0.99 | 0.90 | 1.00 | 0.83 | 0.83 | 0.88 | 0.78 | 0.77 |
|  | 79 | 0.98 | 0.87 | 0.80 | 0.86 | 0.94 | 0.90 | 0.84 | 0.68 |
|  | 80 | 0.89 | 0.93 | 1.00 | 0.87 | 0.96 | 0.91 | 0.76 | 0.66 |
|  | 81 | 0.87 | 0.79 | 1.00 | 0.81 | 0.96 | 0.84 | 0.83 | 0.55 |
|  | 82 | 0.91 | 0.91 | 0.83 | 0.84 | 0.85 | 0.86 | 0.74 | 0.59 |
|  | 83 | 0.94 | 0.71 | 0.80 | 0.91 | 0.95 | 0.90 | 0.67 | 0.62 |
|  | 84 | 0.87 | 0.91 | 1.00 | 0.90 | 0.96 | 0.92 | 0.81 | 0.80 |
|  | 85 | 0.82 | 0.80 | 1.00 | 0.91 | 0.86 | 0.88 | 0.64 | 0.75 |
|  | 86 | 0.96 | 0.94 | 0.80 | 0.90 | 0.88 | 0.86 | 0.74 | 0.62 |
|  | 87 | 0.73 | 0.73 | 0.86 | 0.90 | 0.87 | 0.86 | 0.96 | 0.63 |
|  | 88 | 0.89 | 0.87 | 1.00 | 0.84 | 0.94 | 0.90 | 0.77 | 0.62 |
|  | 89 | 0.95 | 0.84 | 1.00 | 0.87 | 0.94 | 0.85 | 0.71 | 0.80 |
|  | 90 | 0.86 | 0.93 | 0.83 | 0.92 | 0.97 | 0.91 | 0.78 | 0.55 |
|  | 91 | 0.96 | 0.93 | 0.80 | 0.90 | 0.96 | 0.93 | 0.71 | 0.68 |
|  | 92 | 0.95 | 0.88 | 1.00 | 0.87 | 0.82 | 0.88 | 0.64 | 0.65 |
|  | 93 | 0.84 | 0.91 | 0.80 | 0.89 | 0.83 | 0.91 | 0.85 | 0.88 |
|  | 94 | 0.83 | 0.89 | 0.84 | 0.90 | 0.85 | 0.84 | 0.81 | 0.66 |
| Treatment 3 | 1 | 0.65 | 0.61 | 0.80 | 0.55 | 0.73 | 0.56 | 0.44 | 0.51 |
|  | 2 | 0.49 | 0.78 | 1.00 | 0.70 | 0.57 | 0.75 | 0.57 | 0.69 |
|  | 3 | 0.72 | 0.71 | 0.71 | 0.64 | 0.59 | 0.66 | 0.55 | 0.55 |
|  | 4 | 0.72 | 0.61 | 0.80 | 0.67 | 0.82 | 0.74 | 0.55 | 0.60 |
|  | 5 | 0.96 | 0.79 | 1.00 | 0.72 | 0.78 | 0.77 | 0.62 | 0.92 |
|  | 6 | 0.74 | 0.83 | 0.80 | 0.63 | 0.92 | 0.76 | 0.53 | 0.63 |
|  | 7 | 0.88 | 0.85 | 0.83 | 0.63 | 0.82 | 0.78 | 0.56 | 0.59 |
|  | 8 | 0.73 | 0.79 | 0.83 | 0.63 | 0.86 | 0.74 | 0.52 | 0.63 |
|  | 9 | 0.81 | 0.81 | 0.83 | 0.81 | 0.81 | 0.82 | 0.44 | 0.41 |
|  | 10 | 0.78 | 0.84 | 0.83 | 0.79 | 0.87 | 0.81 | 0.42 | 0.44 |
|  | 11 | 0.85 | 0.79 | 0.80 | 0.71 | 0.95 | 0.77 | 0.57 | 0.57 |
|  | 12 | 0.80 | 0.80 | 1.00 | 0.80 | 0.83 | 0.79 | 0.62 | 0.58 |
|  | 13 | 0.93 | 0.73 | 1.00 | 0.74 | 0.78 | 0.76 | 0.51 | 0.57 |
|  | 14 | 0.64 | 0.71 | 0.67 | 0.70 | 0.73 | 0.69 | 0.57 | 0.44 |
|  | 15 | 0.72 | 0.81 | 1.00 | 0.79 | 0.81 | 0.78 | 0.68 | 0.50 |
|  | 16 | 0.76 | 0.82 | 1.00 | 0.76 | 0.67 | 0.76 | 0.60 | 0.46 |
|  | 17 | 0.85 | 0.82 | 0.67 | 0.81 | 0.65 | 0.79 | 0.48 | 0.61 |
|  | 18 | 0.69 | 0.63 | 0.80 | 0.59 | 0.72 | 0.68 | 0.38 | 0.50 |
|  | 19 | 0.68 | 0.72 | 0.67 | 0.70 | 0.79 | 0.69 | 0.55 | 0.38 |
|  | 20 | 0.86 | 0.52 | 0.80 | 0.67 | 0.62 | 0.58 | 0.48 | 0.50 |
|  | 21 | 0.68 | 0.62 | 0.67 | 0.73 | 0.79 | 0.65 | 0.48 | 0.54 |
|  | 22 | 0.90 | 0.73 | 1.00 | 0.72 | 0.71 | 0.73 | 0.32 | 0.58 |
|  | 23 | 0.72 | 0.61 | 0.80 | 0.64 | 0.76 | 0.58 | 0.61 | 0.69 |
|  | 24 | 0.91 | 0.73 | 0.80 | 0.68 | 0.84 | 0.70 | 2.27 | 0.58 |
|  | 25 | 0.78 | 0.80 | 1.00 | 0.74 | 0.94 | 0.78 | 0.48 | 0.83 |
|  | 26 | 0.83 | 0.90 | 1.25 | 0.82 | 0.86 | 0.63 | 0.56 | 0.51 |
|  | 27 | 0.94 | 0.90 | 1.00 | 0.81 | 0.79 | 0.64 | 0.50 | 0.60 |
|  | 28 | 0.77 | 0.71 | 1.00 | 0.65 | 0.89 | 0.72 | 0.46 | 0.62 |
|  | 29 | 0.95 | 0.75 | 1.00 | 0.67 | 0.83 | 0.71 | 0.67 | 0.52 |
|  | 30 | 0.78 | 0.84 | 0.80 | 0.70 | 0.93 | 0.76 | 0.48 | 0.56 |
|  | 31 | 0.78 | 0.82 | 0.67 | 0.77 | 0.81 | 0.78 | 0.51 | 0.66 |
|  | 32 | 0.80 | 0.58 | 0.67 | 0.61 | 0.80 | 0.58 | 0.27 | 0.81 |
|  | 33 | 0.93 | 0.85 | 1.00 | 0.78 | 0.82 | 0.84 | 0.47 | 0.80 |
|  | 34 | 0.79 | 0.86 | 1.00 | 0.67 | 0.97 | 0.76 | 0.70 | 0.82 |
|  | 35 | 0.71 | 0.74 | 0.80 | 0.64 | 0.78 | 0.74 | 0.48 | 0.95 |
|  | 36 | 0.82 | 0.72 | 1.00 | 0.68 | 0.82 | 0.70 | 0.53 | 0.75 |
|  | 37 | 0.75 | 0.75 | 1.00 | 0.71 | 0.80 | 0.75 | 0.55 | 0.98 |
|  | 38 | 0.67 | 0.76 | 0.80 | 0.82 | 0.77 | 0.77 | 0.39 | 1.00 |
|  | 39 | 0.74 | 0.73 | 0.80 | 0.71 | 0.74 | 0.72 | 0.37 | 0.74 |
|  | 40 | 0.88 | 0.86 | 1.00 | 0.68 | 0.93 | 0.85 | 0.48 | 0.89 |
|  | 41 | 0.86 | 0.84 | 1.00 | 0.70 | 0.96 | 0.84 | 0.49 | 0.97 |
|  | 42 | 0.77 | 0.71 | 0.80 | 0.71 | 0.74 | 0.70 | 0.60 | 0.88 |
|  | 43 | 0.83 | 0.66 | 0.83 | 0.71 | 0.79 | 0.69 | 0.53 | 0.73 |
|  | 44 | 0.84 | 0.88 | 0.80 | 0.68 | 0.79 | 0.77 | 0.48 | 0.97 |
|  | 45 | 0.80 | 0.92 | 0.67 | 0.79 | 0.81 | 0.81 | 0.49 | 0.58 |
|  | 46 | 0.92 | 0.86 | 0.80 | 0.76 | 0.93 | 0.75 | 0.56 | 0.76 |
|  | 47 | 0.86 | 0.80 | 0.80 | 0.75 | 0.86 | 0.75 | 0.62 | 0.62 |
|  | 48 | 0.83 | 0.75 | 1.00 | 0.64 | 0.82 | 0.73 | 0.50 | 0.93 |
|  | 49 | 0.83 | 0.70 | 0.80 | 0.69 | 0.86 | 0.70 | 0.55 | 0.83 |
|  | 50 | 0.67 | 0.74 | 0.83 | 0.74 | 0.76 | 0.69 | 0.54 | 0.85 |
|  | 51 | 0.76 | 0.75 | 0.80 | 0.73 | 0.73 | 0.74 | 0.52 | 0.95 |
|  | 52 | 0.67 | 0.76 | 0.80 | 0.71 | 0.66 | 0.73 | 0.63 | 0.62 |
|  | 53 | 0.69 | 0.67 | 1.00 | 0.69 | 0.79 | 0.72 | 0.47 | 0.78 |
|  | 54 | 0.90 | 0.76 | 1.00 | 0.73 | 0.81 | 0.76 | 0.54 | 0.98 |
|  | 55 | 0.59 | 0.76 | 1.00 | 0.78 | 0.80 | 0.80 | 0.57 | 0.94 |
|  | 56 | 0.77 | 0.74 | 1.00 | 0.75 | 0.76 | 0.75 | 0.46 | 0.98 |
|  | 57 | 0.70 | 0.68 | 1.00 | 0.68 | 0.85 | 0.73 | 0.52 | 0.86 |
|  | 58 | 0.79 | 0.78 | 0.71 | 0.74 | 0.84 | 0.78 | 0.60 | 0.86 |
|  | 59 | 0.70 | 0.67 | 1.00 | 0.70 | 0.80 | 0.73 | 0.45 | 0.82 |
|  | 60 | 0.81 | 0.62 | 1.00 | 0.54 | 0.77 | 0.58 | 0.49 | 0.92 |
|  | 61 | 0.95 | 0.79 | 0.80 | 0.84 | 0.80 | 0.76 | 0.60 | 0.84 |
|  | 62 | 0.80 | 0.70 | 0.80 | 0.69 | 0.81 | 0.83 | 0.53 | 0.73 |
|  | 63 | 0.70 | 0.75 | 0.80 | 0.67 | 0.67 | 0.75 | 0.47 | 0.72 |
|  | 64 | 0.81 | 0.88 | 0.80 | 0.72 | 0.83 | 0.84 | 0.55 | 0.70 |
|  | 65 | 0.55 | 0.69 | 0.80 | 0.71 | 0.67 | 0.53 | 0.53 | 0.75 |
|  | 66 | 0.75 | 0.66 | 0.80 | 0.73 | 0.82 | 0.68 | 0.60 | 0.85 |
|  | 67 | 0.72 | 0.78 | 1.00 | 0.74 | 0.68 | 0.78 | 0.64 | 0.80 |
|  | 68 | 0.72 | 0.69 | 0.80 | 0.74 | 0.67 | 0.75 | 0.53 | 0.88 |
|  | 69 | 0.79 | 0.67 | 0.80 | 0.71 | 0.75 | 0.79 | 0.58 | 0.77 |
|  | 70 | 0.70 | 0.69 | 0.80 | 0.68 | 0.79 | 0.75 | 0.58 | 0.58 |
|  | 71 | 0.78 | 0.71 | 1.00 | 0.69 | 0.82 | 0.73 | 0.53 | 0.64 |
|  | 72 | 0.84 | 0.59 | 0.67 | 0.63 | 0.78 | 0.59 | 0.47 | 0.57 |
|  | 73 | 0.69 | 0.75 | 0.80 | 0.67 | 0.58 | 0.72 | 0.51 | 0.80 |
|  | 74 | 0.73 | 0.86 | 1.00 | 0.84 | 0.65 | 0.80 | 0.69 | 0.63 |
|  | 75 | 0.80 | 0.52 | 0.83 | 0.54 | 0.71 | 0.79 | 0.40 | 0.89 |
|  | 76 | 0.68 | 0.79 | 0.80 | 0.69 | 0.65 | 0.75 | 0.54 | 0.69 |
|  | 77 | 0.72 | 0.64 | 0.71 | 0.64 | 0.71 | 0.69 | 0.52 | 0.71 |
|  | 78 | 0.86 | 0.79 | 1.00 | 0.68 | 0.73 | 0.76 | 0.61 | 0.97 |
|  | 79 | 0.84 | 0.76 | 0.80 | 0.74 | 0.80 | 0.79 | 0.66 | 0.96 |
|  | 80 | 0.86 | 0.84 | 0.80 | 0.71 | 0.90 | 0.78 | 0.63 | 0.83 |
|  | 81 | 0.81 | 0.71 | 0.89 | 0.65 | 0.91 | 0.70 | 0.59 | 0.69 |
|  | 82 | 0.86 | 0.78 | 0.67 | 0.68 | 0.77 | 0.71 | 0.52 | 0.71 |
|  | 83 | 0.82 | 0.58 | 0.80 | 0.67 | 0.81 | 0.69 | 0.39 | 0.80 |
|  | 84 | 0.68 | 0.74 | 1.00 | 0.72 | 0.79 | 0.77 | 0.57 | 0.85 |
|  | 85 | 0.70 | 0.75 | 0.80 | 0.79 | 0.80 | 0.68 | 0.48 | 0.81 |
|  | 86 | 0.80 | 0.77 | 0.80 | 0.76 | 0.84 | 0.75 | 0.55 | 0.84 |
|  | 87 | 0.65 | 0.62 | 0.57 | 0.71 | 0.75 | 0.70 | 0.80 | 0.87 |
|  | 88 | 0.84 | 0.76 | 0.80 | 0.74 | 0.95 | 0.76 | 0.55 | 0.83 |
|  | 89 | 0.92 | 0.72 | 0.80 | 0.78 | 0.92 | 0.76 | 0.50 | 0.85 |
|  | 90 | 0.82 | 0.89 | 0.67 | 0.80 | 0.98 | 0.80 | 0.66 | 0.68 |
|  | 91 | 0.93 | 0.83 | 0.80 | 0.81 | 0.91 | 0.79 | 0.50 | 0.93 |
|  | 92 | 0.81 | 0.85 | 1.00 | 0.85 | 0.82 | 0.64 | 0.33 | 0.82 |
|  | 93 | 0.68 | 0.84 | 0.80 | 0.77 | 0.79 | 0.72 | 0.64 | 0.93 |
|  | 94 | 0.93 | 0.79 | 0.72 | 0.76 | 0.84 | 0.75 | 0.55 | 0.87 |
| Treatment 4 | 1 | 0.58 | 0.45 | 0.80 | 0.43 | 0.58 | 0.41 | 0.29 | 0.47 |
|  | 2 | 0.40 | 0.56 | 1.00 | 0.54 | 0.52 | 0.60 | 0.32 | 0.67 |
|  | 3 | 0.65 | 0.58 | 0.57 | 0.48 | 0.51 | 0.54 | 0.40 | 0.39 |
|  | 4 | 0.58 | 0.50 | 0.80 | 0.54 | 0.63 | 0.62 | 0.43 | 0.56 |
|  | 5 | 0.80 | 0.61 | 1.00 | 0.54 | 0.71 | 0.57 | 0.40 | 0.72 |
|  | 6 | 0.67 | 0.62 | 0.80 | 0.52 | 0.71 | 0.63 | 0.18 | 0.49 |
|  | 7 | 0.77 | 0.65 | 0.67 | 0.48 | 0.77 | 0.60 | 0.41 | 0.31 |
|  | 8 | 0.70 | 0.65 | 0.67 | 0.49 | 0.61 | 0.58 | 0.42 | 0.37 |
|  | 9 | 0.71 | 0.72 | 0.67 | 0.66 | 0.78 | 0.70 | 0.35 | 0.21 |
|  | 10 | 0.71 | 0.74 | 0.67 | 0.69 | 0.83 | 0.70 | 0.23 | 0.25 |
|  | 11 | 0.83 | 0.63 | 0.80 | 0.56 | 0.76 | 0.62 | 0.36 | 0.33 |
|  | 12 | 0.69 | 0.75 | 1.00 | 0.71 | 0.70 | 0.72 | 0.37 | 0.37 |
|  | 13 | 0.77 | 0.63 | 1.00 | 0.55 | 0.74 | 0.61 | 0.28 | 0.40 |
|  | 14 | 0.49 | 0.57 | 0.67 | 0.58 | 0.67 | 0.56 | 0.26 | 0.27 |
|  | 15 | 0.69 | 0.70 | 0.80 | 0.69 | 0.60 | 0.69 | 0.39 | 0.33 |
|  | 16 | 0.53 | 0.56 | 1.00 | 0.49 | 0.60 | 0.53 | 0.35 | 0.31 |
|  | 17 | 0.84 | 0.56 | 0.67 | 0.60 | 0.57 | 0.57 | 0.23 | 0.39 |
|  | 18 | 0.51 | 0.53 | 0.80 | 0.46 | 0.54 | 0.52 | 0.24 | 0.33 |
|  | 19 | 0.57 | 0.53 | 0.67 | 0.49 | 0.68 | 0.51 | 0.36 | 0.20 |
|  | 20 | 0.71 | 0.43 | 0.80 | 0.52 | 0.60 | 0.46 | 0.27 | 0.31 |
|  | 21 | 0.49 | 0.51 | 0.67 | 0.52 | 0.49 | 0.51 | 0.30 | 0.28 |
|  | 22 | 0.69 | 0.61 | 1.00 | 0.54 | 0.58 | 0.56 | 0.17 | 0.37 |
|  | 23 | 0.71 | 0.51 | 0.80 | 0.49 | 0.70 | 0.49 | 0.56 | 0.46 |
|  | 24 | 0.90 | 0.68 | 0.80 | 0.52 | 0.76 | 0.58 | 0.22 | 0.45 |
|  | 25 | 0.75 | 0.70 | 1.00 | 0.61 | 0.96 | 0.66 | 0.26 | 0.65 |
|  | 26 | 0.78 | 0.75 | 1.00 | 0.48 | 0.83 | 0.49 | 0.51 | 0.30 |
|  | 27 | 0.89 | 0.77 | 0.80 | 0.63 | 0.81 | 0.48 | 0.29 | 0.40 |
|  | 28 | 0.60 | 0.63 | 1.00 | 0.52 | 0.75 | 0.60 | 0.31 | 0.40 |
|  | 29 | 0.89 | 0.63 | 1.00 | 0.52 | 0.80 | 0.59 | 0.27 | 0.40 |
|  | 30 | 0.72 | 0.76 | 0.80 | 0.58 | 0.93 | 0.63 | 0.16 | 0.44 |
|  | 31 | 0.79 | 0.76 | 0.67 | 0.66 | 0.77 | 0.69 | 0.19 | 0.39 |
|  | 32 | 0.69 | 0.43 | 0.67 | 0.39 | 0.60 | 0.38 | 0.13 | 0.37 |
|  | 33 | 0.86 | 0.77 | 1.00 | 0.67 | 0.79 | 0.71 | 0.27 | 0.39 |
|  | 34 | 0.80 | 0.74 | 1.00 | 0.49 | 0.95 | 0.64 | 0.65 | 0.65 |
|  | 35 | 0.58 | 0.62 | 0.80 | 0.49 | 0.60 | 0.65 | 0.19 | 0.59 |
|  | 36 | 0.70 | 0.59 | 1.00 | 0.53 | 0.59 | 0.57 | 0.30 | 0.46 |
|  | 37 | 0.70 | 0.64 | 1.00 | 0.53 | 0.64 | 0.59 | 0.31 | 0.60 |
|  | 38 | 0.65 | 0.60 | 0.80 | 0.57 | 0.70 | 0.65 | 0.22 | 0.69 |
|  | 39 | 0.61 | 0.62 | 0.60 | 0.57 | 0.57 | 0.58 | 0.24 | 0.44 |
|  | 40 | 0.86 | 0.73 | 1.00 | 0.50 | 0.88 | 0.72 | 0.31 | 0.58 |
|  | 41 | 0.74 | 0.76 | 1.00 | 0.55 | 0.86 | 0.73 | 0.28 | 0.64 |
|  | 42 | 0.70 | 0.53 | 0.80 | 0.45 | 0.62 | 0.49 | 0.37 | 0.64 |
|  | 43 | 0.73 | 0.59 | 0.67 | 0.59 | 0.74 | 0.59 | 0.33 | 0.39 |
|  | 44 | 0.84 | 0.82 | 0.80 | 0.50 | 0.68 | 0.63 | 0.89 | 0.70 |
|  | 45 | 0.66 | 0.86 | 0.67 | 0.60 | 0.75 | 0.68 | 0.28 | 0.42 |
|  | 46 | 0.79 | 0.77 | 0.80 | 0.55 | 0.86 | 0.59 | 0.29 | 0.49 |
|  | 47 | 0.83 | 0.70 | 0.80 | 0.54 | 0.66 | 0.67 | 0.40 | 0.40 |
|  | 48 | 0.67 | 0.65 | 1.00 | 0.54 | 0.69 | 0.57 | 0.30 | 0.89 |
|  | 49 | 0.67 | 0.55 | 0.80 | 0.44 | 0.72 | 0.50 | 0.32 | 0.52 |
|  | 50 | 0.63 | 0.62 | 0.83 | 0.61 | 0.64 | 0.60 | 0.38 | 0.46 |
|  | 51 | 0.62 | 0.60 | 0.80 | 0.60 | 0.69 | 0.61 | 0.30 | 0.72 |
|  | 52 | 0.57 | 0.62 | 0.80 | 0.58 | 0.51 | 0.59 | 0.41 | 0.38 |
|  | 53 | 0.57 | 0.56 | 1.00 | 0.46 | 0.63 | 0.52 | 0.24 | 0.44 |
|  | 54 | 0.75 | 0.67 | 1.00 | 0.50 | 0.65 | 0.59 | 0.22 | 0.57 |
|  | 55 | 0.52 | 0.61 | 1.00 | 0.62 | 0.70 | 0.65 | 0.38 | 0.50 |
|  | 56 | 0.70 | 0.45 | 1.00 | 0.46 | 0.64 | 0.43 | 0.44 | 0.60 |
|  | 57 | 0.54 | 0.55 | 1.00 | 0.52 | 0.51 | 0.58 | 0.33 | 0.49 |
|  | 58 | 0.71 | 0.71 | 0.71 | 0.60 | 0.79 | 0.70 | 0.44 | 0.46 |
|  | 59 | 0.58 | 0.57 | 1.00 | 0.55 | 0.76 | 0.60 | 0.17 | 0.43 |
|  | 60 | 0.66 | 0.46 | 1.00 | 0.42 | 0.64 | 0.43 | 0.23 | 0.59 |
|  | 61 | 0.62 | 0.67 | 0.80 | 0.68 | 0.73 | 0.64 | 0.35 | 0.44 |
|  | 62 | 0.70 | 0.58 | 0.80 | 0.50 | 0.66 | 0.61 | 0.34 | 0.34 |
|  | 63 | 0.67 | 0.69 | 0.80 | 0.54 | 0.62 | 0.64 | 0.30 | 0.42 |
|  | 64 | 0.69 | 0.79 | 0.80 | 0.54 | 0.69 | 0.76 | 0.31 | 0.46 |
|  | 65 | 0.89 | 0.57 | 0.80 | 0.53 | 0.81 | 0.41 | 0.35 | 0.57 |
|  | 66 | 0.69 | 0.55 | 0.80 | 0.56 | 0.69 | 0.56 | 0.39 | 0.42 |
|  | 67 | 0.67 | 0.69 | 1.00 | 0.61 | 0.65 | 0.67 | 0.39 | 0.46 |
|  | 68 | 0.58 | 0.50 | 0.80 | 0.44 | 0.63 | 0.52 | 0.29 | 0.60 |
|  | 69 | 0.65 | 0.51 | 0.80 | 0.52 | 0.69 | 0.58 | 0.34 | 0.53 |
|  | 70 | 0.53 | 0.56 | 0.80 | 0.45 | 0.74 | 0.60 | 0.38 | 0.36 |
|  | 71 | 0.71 | 0.59 | 1.00 | 0.49 | 0.66 | 0.59 | 0.32 | 0.37 |
|  | 72 | 0.69 | 0.39 | 0.67 | 0.43 | 0.59 | 0.40 | 0.25 | 0.37 |
|  | 73 | 0.50 | 0.53 | 0.80 | 0.51 | 0.50 | 0.54 | 0.36 | 0.47 |
|  | 74 | 0.65 | 0.66 | 0.80 | 0.64 | 0.61 | 0.65 | 0.42 | 0.40 |
|  | 75 | 0.44 | 0.32 | 0.67 | 0.33 | 0.40 | 0.45 | 0.14 | 0.45 |
|  | 76 | 0.60 | 0.47 | 0.80 | 0.51 | 0.60 | 0.52 | 0.25 | 0.45 |
|  | 77 | 0.68 | 0.47 | 0.71 | 0.48 | 0.59 | 0.49 | 0.25 | 0.43 |
|  | 78 | 0.56 | 0.63 | 1.00 | 0.47 | 0.60 | 0.56 | 0.41 | 0.76 |
|  | 79 | 0.63 | 0.60 | 0.80 | 0.63 | 0.75 | 0.67 | 0.37 | 0.49 |
|  | 80 | 0.82 | 0.73 | 0.80 | 0.66 | 0.86 | 0.68 | 0.44 | 0.55 |
|  | 81 | 0.79 | 0.62 | 0.83 | 0.54 | 0.81 | 0.61 | 0.44 | 0.33 |
|  | 82 | 0.75 | 0.61 | 0.67 | 0.54 | 0.65 | 0.61 | 0.22 | 0.47 |
|  | 83 | 0.75 | 0.43 | 0.80 | 0.47 | 0.71 | 0.52 | 0.24 | 0.46 |
|  | 84 | 0.64 | 0.63 | 1.00 | 0.55 | 0.70 | 0.63 | 0.38 | 0.67 |
|  | 85 | 0.65 | 0.39 | 0.80 | 0.46 | 0.67 | 0.44 | 0.31 | 0.65 |
|  | 86 | 0.79 | 0.71 | 0.80 | 0.58 | 0.82 | 0.61 | 0.37 | 0.41 |
|  | 87 | 0.58 | 0.45 | 0.57 | 0.54 | 0.73 | 0.53 | 0.43 | 0.46 |
|  | 88 | 0.81 | 0.67 | 0.80 | 0.63 | 0.83 | 0.70 | 0.41 | 0.44 |
|  | 89 | 0.85 | 0.59 | 0.80 | 0.64 | 0.80 | 0.63 | 0.35 | 0.65 |
|  | 90 | 0.73 | 0.79 | 0.67 | 0.68 | 0.81 | 0.68 | 0.46 | 0.36 |
|  | 91 | 0.79 | 0.81 | 0.80 | 0.70 | 0.86 | 0.66 | 0.37 | 0.55 |
|  | 92 | 0.83 | 0.73 | 1.00 | 0.61 | 0.70 | 0.42 | 0.23 | 0.50 |
|  | 93 | 0.67 | 0.38 | 0.80 | 0.40 | 0.68 | 0.47 | 0.29 | 0.82 |
|  | 94 | 0.82 | 0.71 | 0.60 | 0.62 | 0.81 | 0.63 | 0.36 | 0.51 |
| Mean of all treatments | 1 | 0.75 | 0.72 | 0.85 | 0.71 | 0.77 | 0.70 | 0.58 | 0.22 |
|  | 2 | 0.63 | 0.79 | 1.00 | 0.77 | 0.71 | 0.80 | 0.65 | 0.33 |
|  | 3 | 0.78 | 0.79 | 0.79 | 0.73 | 0.71 | 0.75 | 0.67 | 0.23 |
|  | 4 | 0.80 | 0.69 | 0.90 | 0.75 | 0.83 | 0.80 | 0.68 | 0.33 |
|  | 5 | 0.94 | 0.82 | 1.00 | 0.77 | 0.86 | 0.80 | 0.72 | 0.36 |
|  | 6 | 0.84 | 0.82 | 0.87 | 0.75 | 0.90 | 0.81 | 0.61 | 0.26 |
|  | 7 | 0.90 | 0.84 | 0.88 | 0.70 | 0.86 | 0.81 | 0.69 | 0.16 |
|  | 8 | 0.83 | 0.85 | 0.83 | 0.75 | 0.87 | 0.80 | 0.68 | 0.29 |
|  | 9 | 0.87 | 0.85 | 0.83 | 0.85 | 0.89 | 0.86 | 0.60 | 0.07 |
|  | 10 | 0.86 | 0.88 | 0.83 | 0.85 | 0.94 | 0.85 | 0.59 | 0.13 |
|  | 11 | 0.92 | 0.84 | 0.85 | 0.80 | 0.93 | 0.84 | 0.67 | 0.13 |
|  | 12 | 0.84 | 0.87 | 1.00 | 0.84 | 0.87 | 0.85 | 0.70 | 0.17 |
|  | 13 | 0.95 | 0.81 | 1.00 | 0.79 | 0.85 | 0.81 | 0.62 | 0.24 |
|  | 14 | 0.72 | 0.77 | 0.79 | 0.77 | 0.80 | 0.75 | 0.64 | 0.14 |
|  | 15 | 0.81 | 0.86 | 0.95 | 0.85 | 0.82 | 0.85 | 0.74 | 0.15 |
|  | 16 | 0.79 | 0.80 | 1.00 | 0.77 | 0.77 | 0.78 | 0.69 | 0.13 |
|  | 17 | 0.89 | 0.83 | 0.79 | 0.84 | 0.76 | 0.83 | 0.61 | 0.22 |
|  | 18 | 0.77 | 0.75 | 0.85 | 0.71 | 0.80 | 0.76 | 0.56 | 0.13 |
|  | 19 | 0.78 | 0.78 | 0.79 | 0.75 | 0.86 | 0.75 | 0.67 | 0.12 |
|  | 20 | 0.89 | 0.69 | 0.90 | 0.75 | 0.76 | 0.71 | 0.61 | 0.14 |
|  | 21 | 0.74 | 0.76 | 0.79 | 0.77 | 0.78 | 0.76 | 0.63 | 0.07 |
|  | 22 | 0.89 | 0.81 | 1.00 | 0.79 | 0.81 | 0.80 | 0.53 | 0.18 |
|  | 23 | 0.85 | 0.72 | 0.85 | 0.72 | 0.82 | 0.72 | 0.70 | 0.23 |
|  | 24 | 0.94 | 0.83 | 0.90 | 0.76 | 0.90 | 0.80 | 1.07 | 0.18 |
|  | 25 | 0.87 | 0.87 | 1.00 | 0.80 | 0.95 | 0.83 | 0.59 | 0.22 |
|  | 26 | 0.90 | 0.90 | 1.13 | 0.81 | 0.92 | 0.74 | 0.74 | 0.15 |
|  | 27 | 0.95 | 0.90 | 1.00 | 0.84 | 0.86 | 0.74 | 0.61 | 0.14 |
|  | 28 | 0.79 | 0.81 | 1.00 | 0.76 | 0.90 | 0.80 | 0.64 | 0.17 |
|  | 29 | 0.95 | 0.82 | 1.00 | 0.76 | 0.91 | 0.80 | 0.92 | 0.21 |
|  | 30 | 0.87 | 0.88 | 0.85 | 0.78 | 0.94 | 0.82 | 0.60 | 0.19 |
|  | 31 | 0.87 | 0.89 | 0.79 | 0.83 | 0.86 | 0.85 | 0.63 | 0.19 |
|  | 32 | 0.86 | 0.71 | 0.79 | 0.72 | 0.84 | 0.69 | 0.48 | 0.17 |
|  | 33 | 0.94 | 0.89 | 1.00 | 0.84 | 0.86 | 0.87 | 0.61 | 0.32 |
|  | 34 | 0.90 | 0.88 | 1.00 | 0.75 | 0.96 | 0.82 | 0.68 | 0.32 |
|  | 35 | 0.79 | 0.80 | 0.85 | 0.74 | 0.80 | 0.81 | 0.60 | 0.29 |
|  | 36 | 0.84 | 0.79 | 1.00 | 0.77 | 0.84 | 0.77 | 0.66 | 0.21 |
|  | 37 | 0.85 | 0.81 | 1.00 | 0.78 | 0.83 | 0.80 | 0.65 | 0.24 |
|  | 38 | 0.80 | 0.82 | 0.85 | 0.82 | 0.86 | 0.84 | 0.58 | 0.33 |
|  | 39 | 0.79 | 0.81 | 0.85 | 0.79 | 0.79 | 0.80 | 0.57 | 0.20 |
|  | 40 | 0.92 | 0.88 | 1.00 | 0.76 | 0.94 | 0.88 | 0.60 | 0.27 |
|  | 41 | 0.88 | 0.88 | 1.00 | 0.78 | 0.94 | 0.88 | 0.65 | 0.31 |
|  | 42 | 0.84 | 0.77 | 0.85 | 0.77 | 0.81 | 0.77 | 0.69 | 0.29 |
|  | 43 | 0.85 | 0.77 | 0.88 | 0.78 | 0.86 | 0.78 | 0.66 | 0.17 |
|  | 44 | 0.89 | 0.91 | 0.85 | 0.77 | 0.83 | 0.83 | 0.78 | 0.35 |
|  | 45 | 0.83 | 0.93 | 0.79 | 0.83 | 0.87 | 0.85 | 0.61 | 0.21 |
|  | 46 | 0.91 | 0.90 | 0.85 | 0.80 | 0.93 | 0.81 | 0.65 | 0.16 |
|  | 47 | 0.90 | 0.84 | 0.90 | 0.79 | 0.85 | 0.82 | 0.70 | 0.19 |
|  | 48 | 0.85 | 0.84 | 1.00 | 0.75 | 0.86 | 0.79 | 0.65 | 0.41 |
|  | 49 | 0.85 | 0.76 | 0.90 | 0.76 | 0.88 | 0.76 | 0.66 | 0.29 |
|  | 50 | 0.77 | 0.81 | 0.92 | 0.81 | 0.81 | 0.79 | 0.67 | 0.21 |
|  | 51 | 0.82 | 0.80 | 0.85 | 0.82 | 0.83 | 0.81 | 0.63 | 0.35 |
|  | 52 | 0.76 | 0.81 | 0.85 | 0.78 | 0.75 | 0.80 | 0.70 | 0.20 |
|  | 53 | 0.77 | 0.77 | 1.00 | 0.76 | 0.83 | 0.78 | 0.60 | 0.22 |
|  | 54 | 0.91 | 0.82 | 1.00 | 0.79 | 0.83 | 0.81 | 0.64 | 0.26 |
|  | 55 | 0.72 | 0.81 | 1.00 | 0.83 | 0.84 | 0.84 | 0.68 | 0.23 |
|  | 56 | 0.85 | 0.76 | 1.00 | 0.80 | 0.82 | 0.77 | 0.65 | 0.30 |
|  | 57 | 0.77 | 0.76 | 1.00 | 0.77 | 0.81 | 0.80 | 0.66 | 0.25 |
|  | 58 | 0.84 | 0.83 | 0.82 | 0.79 | 0.90 | 0.84 | 0.70 | 0.20 |
|  | 59 | 0.78 | 0.78 | 1.00 | 0.78 | 0.88 | 0.81 | 0.57 | 0.20 |
|  | 60 | 0.84 | 0.70 | 1.00 | 0.69 | 0.81 | 0.71 | 0.61 | 0.28 |
|  | 61 | 0.88 | 0.84 | 0.85 | 0.86 | 0.87 | 0.83 | 0.69 | 0.27 |
|  | 62 | 0.83 | 0.78 | 0.85 | 0.76 | 0.86 | 0.66 | 0.65 | 0.19 |
|  | 63 | 0.80 | 0.82 | 0.90 | 0.76 | 0.79 | 0.81 | 0.60 | 0.20 |
|  | 64 | 0.86 | 0.90 | 0.90 | 0.77 | 0.85 | 0.88 | 0.65 | 0.23 |
|  | 65 | 0.79 | 0.77 | 0.90 | 0.78 | 0.83 | 0.66 | 0.67 | 0.26 |
|  | 66 | 0.83 | 0.76 | 0.90 | 0.79 | 0.87 | 0.77 | 0.70 | 0.18 |
|  | 67 | 0.81 | 0.83 | 1.00 | 0.80 | 0.83 | 0.60 | 0.71 | 0.17 |
|  | 68 | 0.78 | 0.77 | 0.90 | 0.75 | 0.78 | 0.79 | 0.64 | 0.30 |
|  | 69 | 0.82 | 0.75 | 0.85 | 0.78 | 0.84 | 0.81 | 0.68 | 0.26 |
|  | 70 | 0.76 | 0.78 | 0.85 | 0.73 | 0.87 | 0.80 | 0.68 | 0.18 |
|  | 71 | 0.85 | 0.78 | 1.00 | 0.75 | 0.84 | 0.79 | 0.66 | 0.17 |
|  | 72 | 0.87 | 0.70 | 0.79 | 0.72 | 0.83 | 0.69 | 0.62 | 0.19 |
|  | 73 | 0.76 | 0.80 | 0.85 | 0.76 | 0.73 | 0.79 | 0.65 | 0.17 |
|  | 74 | 0.80 | 0.86 | 0.95 | 0.84 | 0.77 | 0.83 | 0.73 | 0.20 |
|  | 75 | 0.79 | 0.65 | 0.83 | 0.68 | 0.76 | 0.83 | 0.56 | 0.20 |
|  | 76 | 0.77 | 0.79 | 0.90 | 0.76 | 0.78 | 0.79 | 0.63 | 0.22 |
|  | 77 | 0.81 | 0.73 | 0.82 | 0.74 | 0.79 | 0.76 | 0.64 | 0.21 |
|  | 78 | 0.85 | 0.83 | 1.00 | 0.75 | 0.79 | 0.80 | 0.70 | 0.35 |
|  | 79 | 0.86 | 0.81 | 0.85 | 0.81 | 0.87 | 0.84 | 0.72 | 0.25 |
|  | 80 | 0.89 | 0.88 | 0.90 | 0.81 | 0.93 | 0.84 | 0.71 | 0.26 |
|  | 81 | 0.87 | 0.78 | 0.93 | 0.75 | 0.92 | 0.79 | 0.71 | 0.15 |
|  | 82 | 0.88 | 0.83 | 0.79 | 0.77 | 0.82 | 0.80 | 0.62 | 0.20 |
|  | 83 | 0.88 | 0.92 | 0.85 | 0.76 | 0.87 | 0.78 | 0.57 | 0.22 |
|  | 84 | 0.80 | 0.82 | 1.00 | 0.79 | 0.86 | 0.83 | 0.69 | 0.30 |
|  | 85 | 0.79 | 0.74 | 0.90 | 0.79 | 0.83 | 0.75 | 0.61 | 0.27 |
|  | 86 | 0.89 | 0.85 | 0.85 | 0.81 | 0.89 | 0.81 | 0.67 | 0.21 |
|  | 87 | 0.74 | 0.70 | 0.75 | 0.79 | 0.84 | 0.46 | 0.80 | 0.19 |
|  | 88 | 0.89 | 0.82 | 0.90 | 0.80 | 0.93 | 0.84 | 0.68 | 0.20 |
|  | 89 | 0.93 | 0.79 | 0.90 | 0.82 | 0.91 | 0.81 | 0.64 | 0.38 |
|  | 90 | 0.85 | 0.90 | 0.79 | 0.85 | 0.94 | 0.85 | 0.72 | 0.17 |
|  | 91 | 0.92 | 0.89 | 0.85 | 0.85 | 0.93 | 0.85 | 0.65 | 0.26 |
|  | 92 | 0.90 | 0.87 | 1.00 | 0.83 | 0.83 | 0.74 | 0.55 | 0.26 |
|  | 93 | 0.80 | 0.78 | 0.85 | 0.76 | 0.82 | 0.78 | 0.69 | 0.38 |
|  | 94 | 0.90 | 0.85 | 0.79 | 0.82 | 0.87 | 0.81 | 0.68 | 0.26 |
