# Supplementary material for: Genome wide association studies reveal candidate genes for salt tolerance in safflower (Carthamus tinctorius L.) at seedling stage
Source: Front Plant Sci. 2026 Mar 6;17:1630492. doi: 10.3389/fpls.2026.1630492 (PMC13003225; doi:10.3389/fpls.2026.1630492)
Supplement: Supplementary Table 7 — Principal component analysis revealed variability among the three groups, based on mean values for PH, FSW, RL, and BY. [file Table7.docx]

**Supplementary Table 7.** Principal component analysis revealed variability among the three groups, based on mean values for PH, FSW, RL, and BY.

| Group | PH | FSW | NL | FRW | RL | BY | DSW | DRW |
| --- | --- | --- | --- | --- | --- | --- | --- | --- |
| Group 1 | 4.86 | 0.53 | 4.52 | 0.32 | 4.01 | 0.92 | 0.14 | 0.12 |
| Group 2 | 4.48 | 0.37 | 4.37 | 0.30 | 3.72 | 0.80 | 0.13 | 0.10 |
| Group 3 | 4.94 | 0.41 | 4.57 | 0.32 | 4.08 | 0.73 | 0.17 | 0.09 |

PH = Plant height, FSW = Fresh shoot weight, NL = Number of leaves, FRW = Fresh root weight, RL = Root length, BY = Biological yield, DSW = Dry shoot weight, DRW = Dry root weight.
