# Supplementary figures and images for: Genome wide association studies reveal candidate genes for salt tolerance in safflower (Carthamus tinctorius L.) at seedling stage
Source: Front Plant Sci. 2026 Mar 6;17:1630492. doi: 10.3389/fpls.2026.1630492 (PMC13003225; doi:10.3389/fpls.2026.1630492)

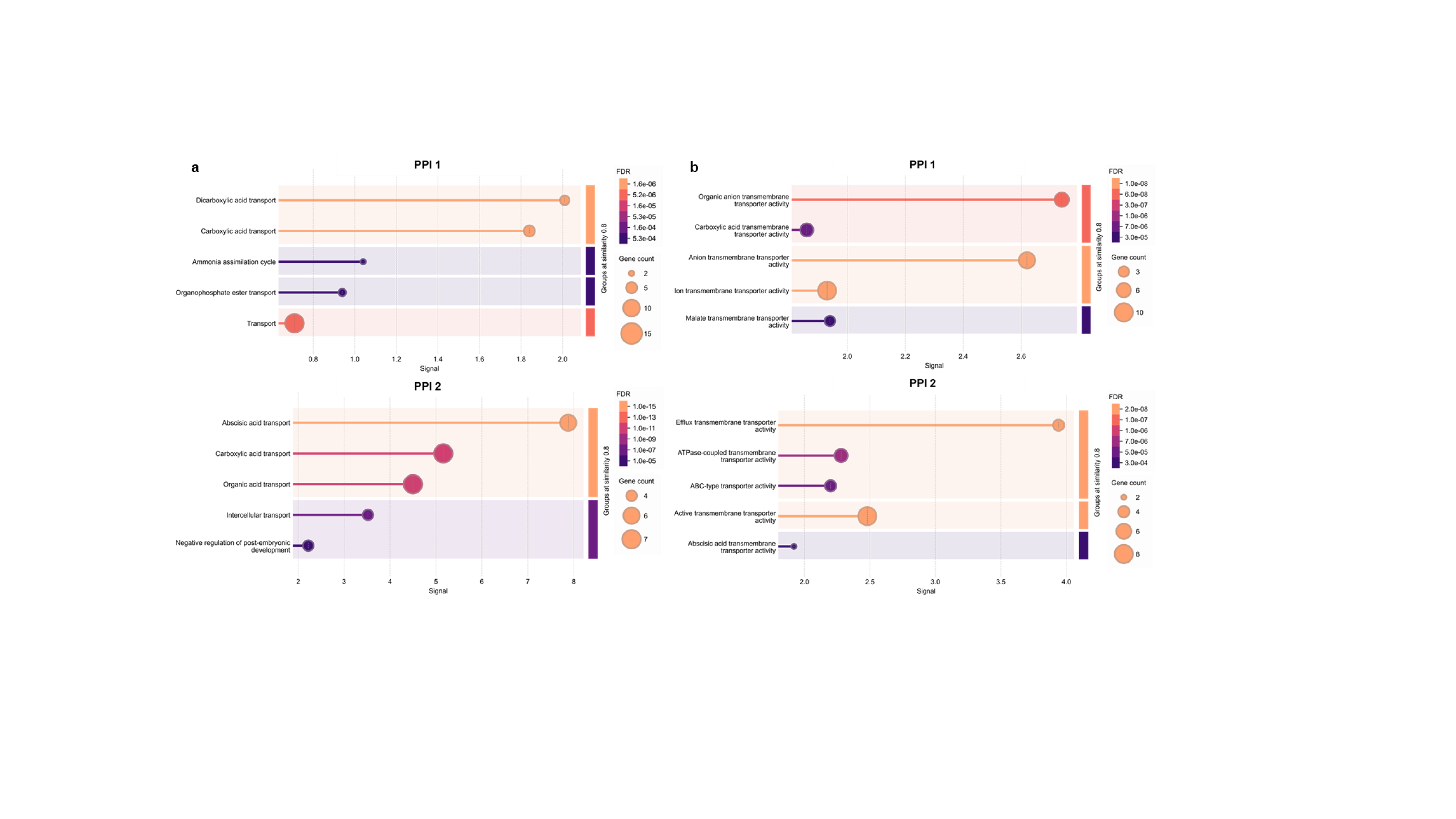

Supplement: Supplementary file 8 [file Image1.tif]

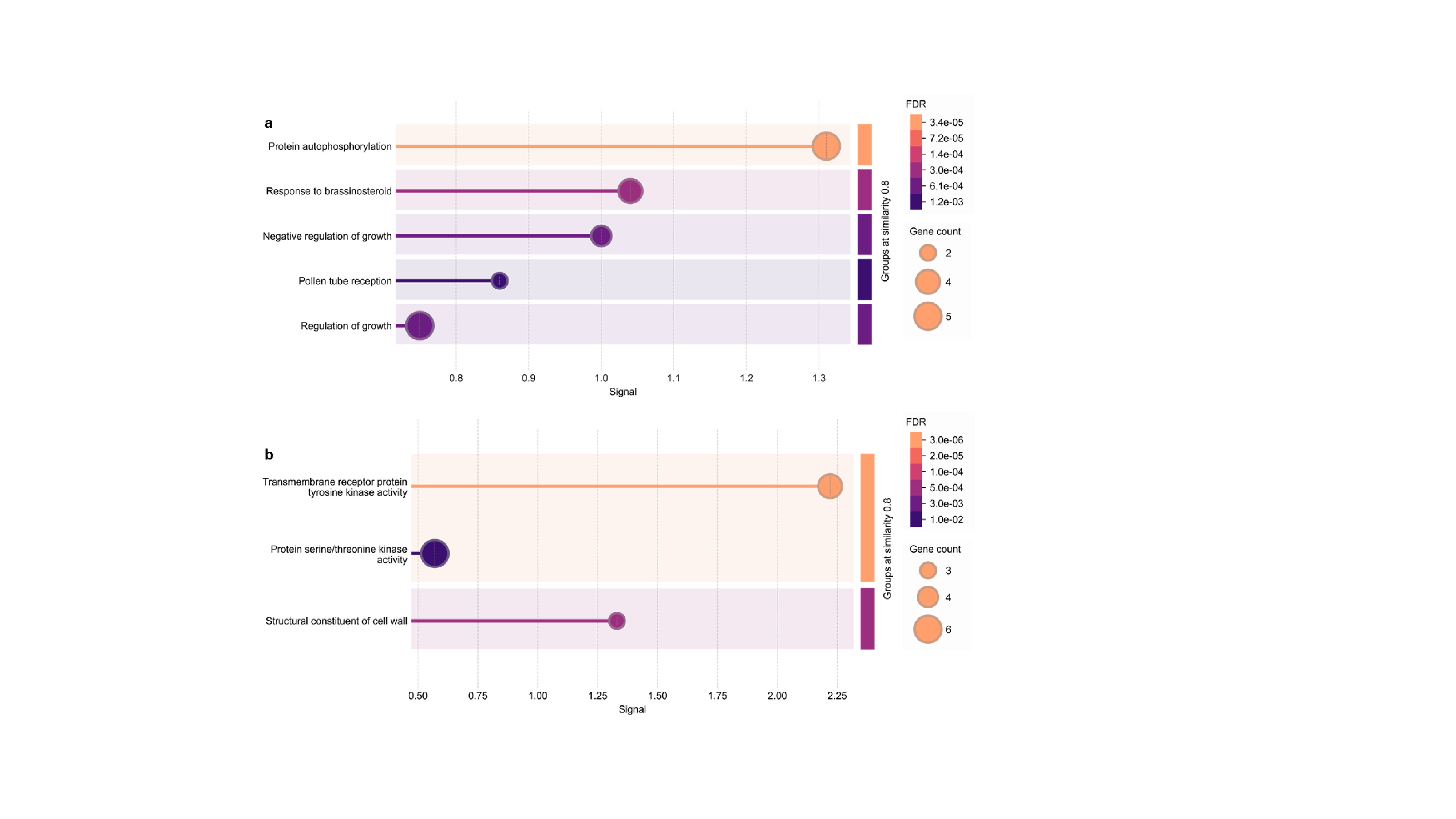

Supplement: Supplementary file 9 [file Image2.tif]

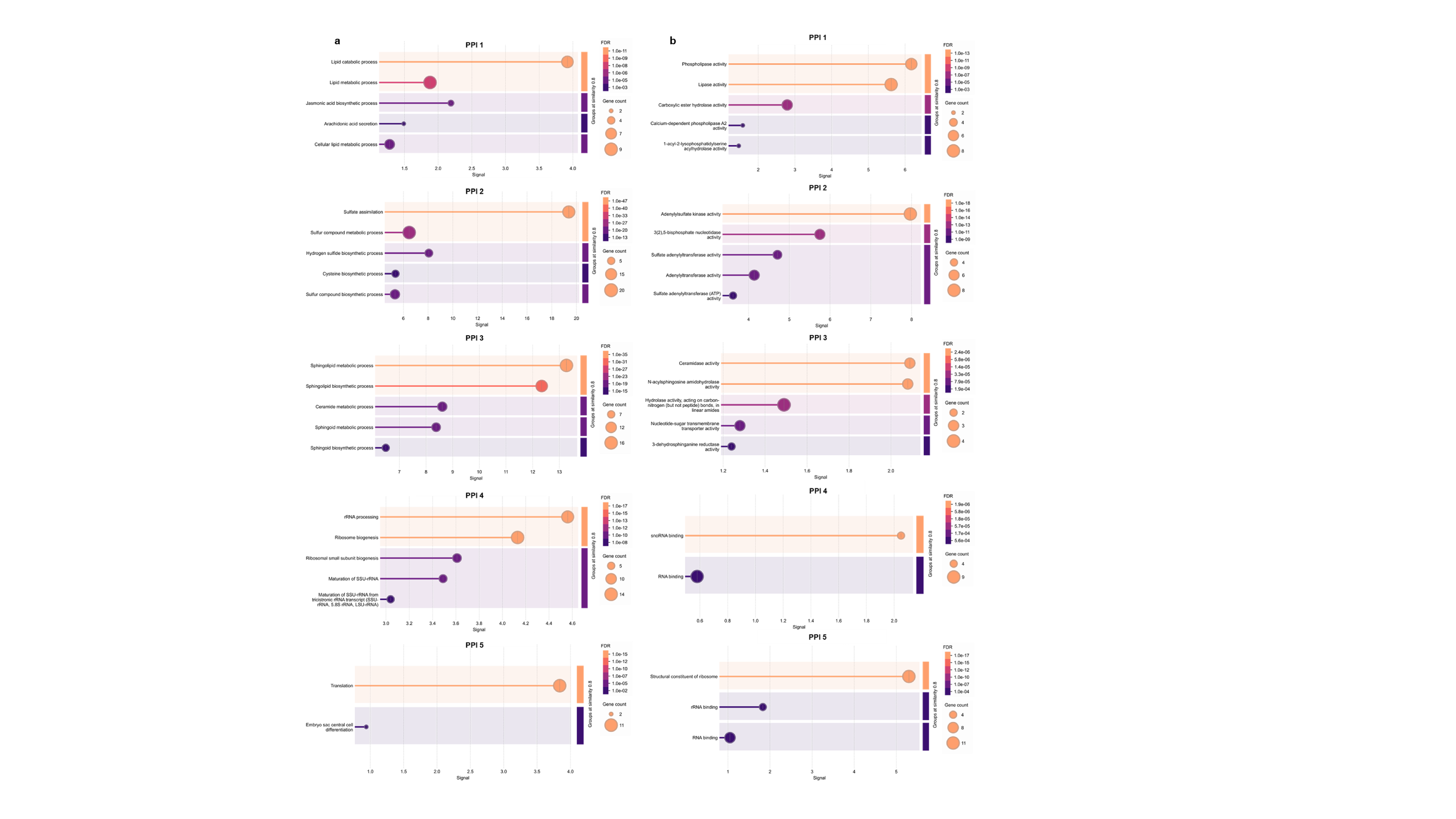

Supplement: Supplementary file 10 [file Image3.tif]

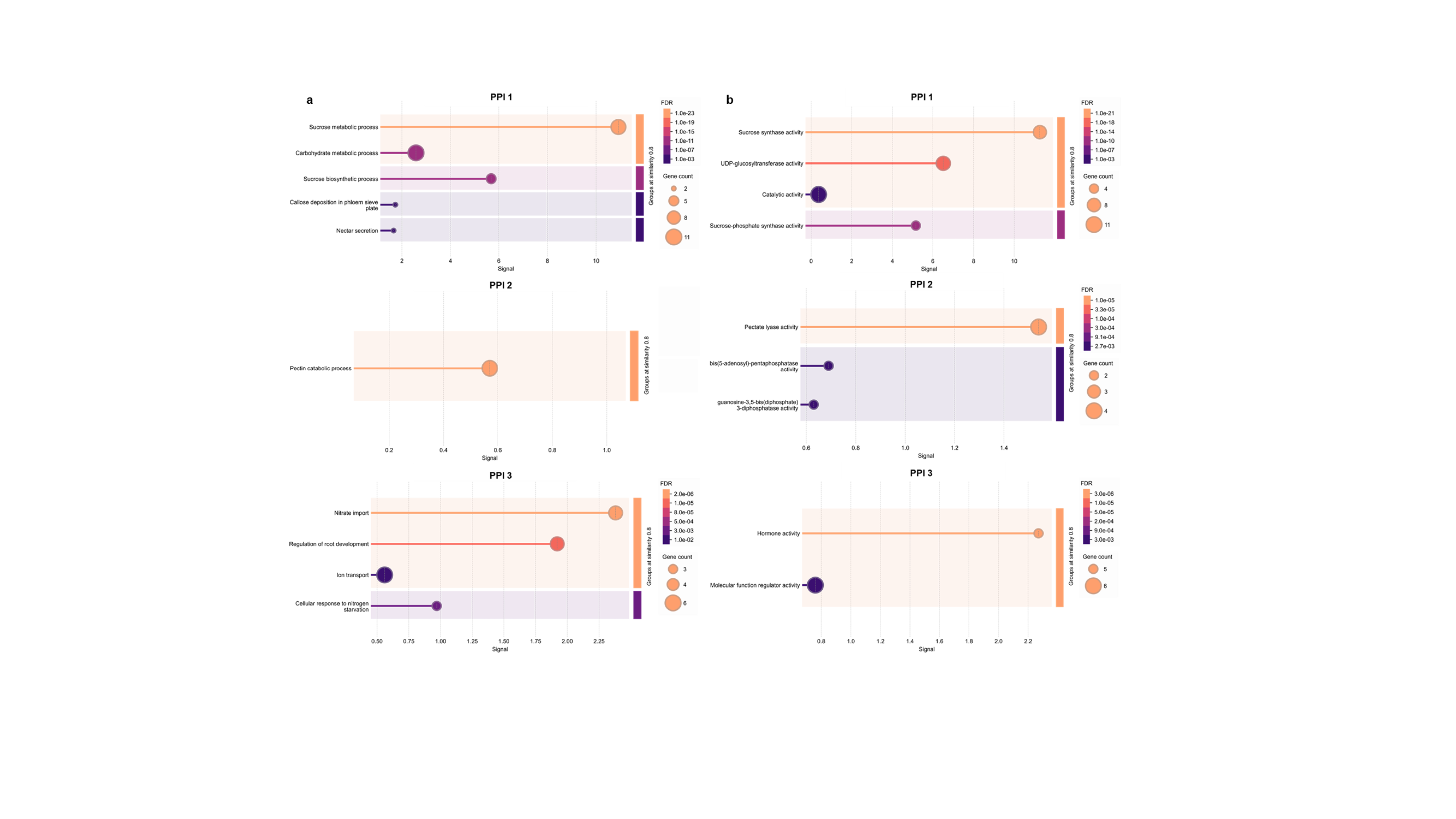

Supplement: Supplementary file 11 [file Image4.tif]

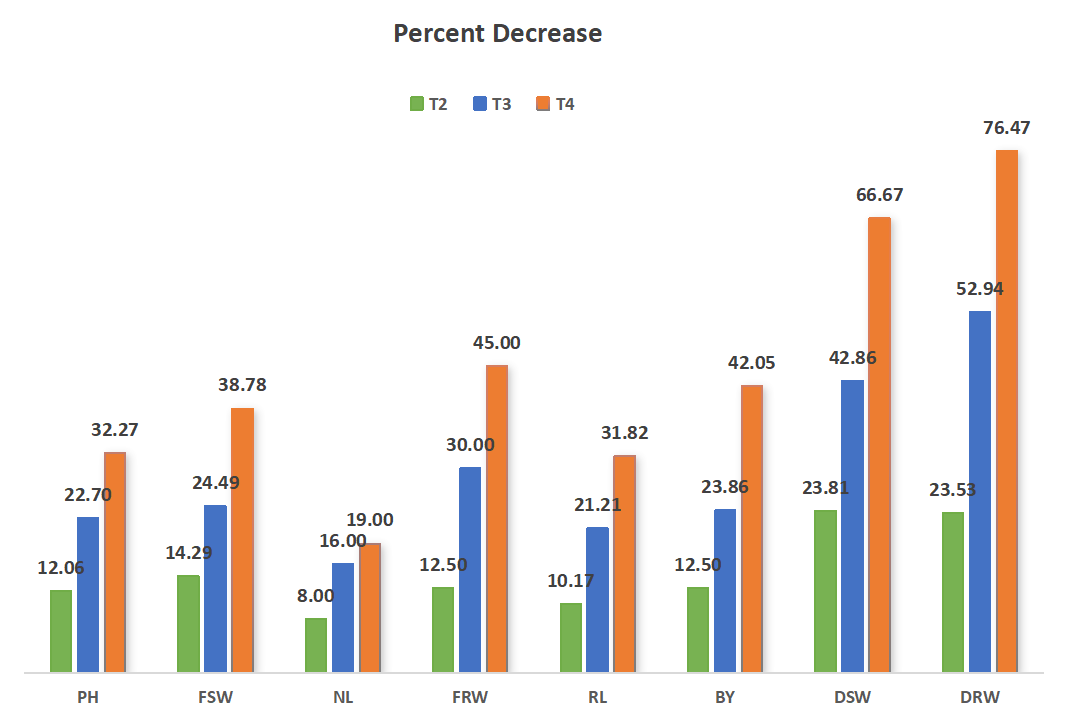

Supplement: Supplementary file 12 [file Image5.png]
